# Material Suitability Testing for Nonmedical Grade Community Face Masks to Decrease Viral Transmission During a Pandemic

**DOI:** 10.1017/dmp.2020.262

**Published:** 2020-07-27

**Authors:** Csanad Varallyay, Ningcheng (Peter) Li, Brendan Case, Bryan Wolf

**Affiliations:** Department of Diagnostic Radiology, Oregon Health & Science University, Portland, Oregon

**Keywords:** communicable diseases, emergency preparedness, infection control, occupational exposure, pandemics

## Abstract

**Objectives:**

Cloth face covering has been recommended by the Centers for Disease Control and Prevention to decrease community viral transmission. This study aims to determine the filtration efficiency and airflow resistance of common household materials available for homemade mask production by comparing numbers of fabrics, various layers, and manipulation.

**Methods:**

Common household woven, knitted, and nonwoven fabrics were tested for filtration efficiency using a fit testing setup and airflow resistance with pressure gauge setup. Three different levels of layering (1, 2, and 4) were tested. Some fabric material was further tested after washing and drying. Filtration performance, the area under the fitted curve comparing airflow resistance and filtration efficiency, was calculated for each fabric material and compared.

**Results:**

Layering increased filtration efficiency and airflow resistance (*P* < 0.0001 and *P* < 0.01, respectively). Polyester felt demonstrated the highest filtration performance index (*P* < 0.0001), higher than all tested 100% cotton materials (all *P* < 0.05) as well as surgical masks (*P* < 0.05). Washing plus drying did not alter filtration performance significantly (*P* > 0.05).

**Conclusions:**

A filtration performance of common household fabrics were compared. Homemade mask designers and producers will have improved data to better balance effectiveness, availability, and comfort with the goal of decreasing community viral transmission.

The novel coronavirus disease 2019 (COVID-19) pandemic globally resulted in 8,351,427 confirmed cases and 449,027 deaths by June 18, 2020.^1^ As evidence of community spread by means of asymptomatic or oligo-symptomatic transmission grew^2–5^ and effectiveness of surgical masks in transmission prevention was better understood,^6^ the Centers for Disease Control and Prevention (CDC) revised its recommendations. They suggest wearing a cloth face covering in public settings where other social distancing measures are difficult to maintain.^7^ Exceptions are people with respiratory difficulty and those who cannot remove a face covering without assistance, including those less than 2 y old.^8^

Given the shortage of medical-grade surgical masks and N95 respirators, homemade masks are recommended for community use.^7^ Prior literature has demonstrated that both professional masks and homemade masks significantly decrease viral exposure and infection risk on a population level.^9^ Davies et al. analyzed readily available materials and concluded that homemade masks made from 100% cotton shirts or pillowcases may serve as effective alternatives during commercial mask shortages.^10^ A main criticism of homemade masks arises from a 2015 randomized control trial, which found higher rates of viral respiratory infection among health-care providers (HCPs) using cloth masks than those using surgical masks^11^; however, the lack of an adequate, non–mask-wearing control arm and less than 60% compliance rates raise questions about their conclusion’s rationality. Regardless, particle filtration efficiency and airflow resistance data of common household materials, especially with different levels of layering (a common mask making technique) and potential washability, remain scarce. Without clear guidance, nonmedical mask designers can only select fabrics based on availability and comfort.^7^

This study aims to provide detailed filtration efficiency and airflow resistance of household materials potentially suitable for homemade masks, which designers and producers can use to better balance effectiveness and comfort. The general public will also be better informed on the properties of nonmedical masks, which may lead to improved designs and higher compliance rates. Although this study does not attempt to define an ideal use for these data, nor does it suggest a standardized mask design, with contoured designs and optimized filtration parameters directing airflow through the mask material, both source control and personal protective applications are reasonable uses in times of commercial mask shortages.

## METHODS

**FIGURE 1:**
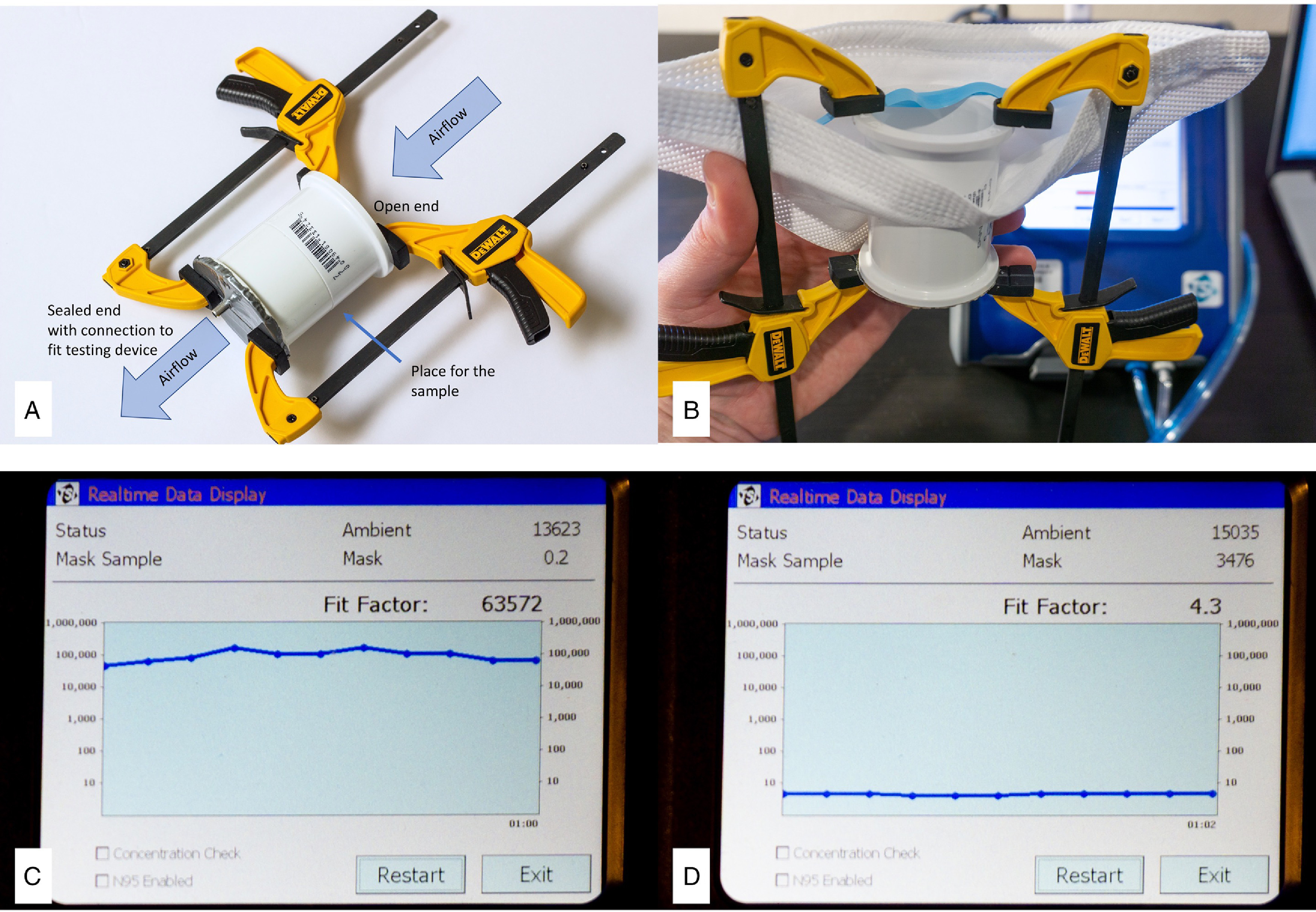
Testing Filtration Efficiency Using PortaCount Pro Plus Respirator Fit Testing Device (TSI; Shoreview, MN). A,B, Each fabric sample was compressed between 2 rigid PVC cylinders, with 4.2 cm inner diameter. One of the cylinders was closed on one end and connected to the measurement line of the fit testing device through a small cannula. Based on measured particle counts, filtration efficiency was calculated to be 100.00% (C, N95 mask) and 76.88% (D, fabric sample).

Filtration efficiency was measured using a quantitative fit testing device (TSI, PortaCount Pro Plus. Shoreview, MN) with a standard 40-nm median diameter particle generator (TSI, Model 8026, Shoreview, MN). This equipment is used routinely for N95 respirator fit testing in clinical settings. Fabric samples were compressed between 2 rigid PVC cylinders, with 4.2-cm inner diameter. One of the cylinders was perfectly sealed on one end and connected to the measurement line of the fit testing device through a small cannula (Figure 1). This is a similar approach to “zero testing” the device using the provided HEPA filter (zero filter) for quality control. During testing, the device creates a negative pressure and draws air through the tested material. Particle concentration was recorded at 1 min of measurement outside (designated as “ambient”) and within the filtered compartment (designated as “filtered”). Three measurements were repeated for each fabric sample. Mean and standard errors of the mean (SEM) were calculated. Filtration efficiency was calculated using the formula:

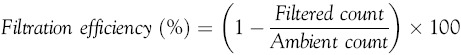


**FIGURE 2:**
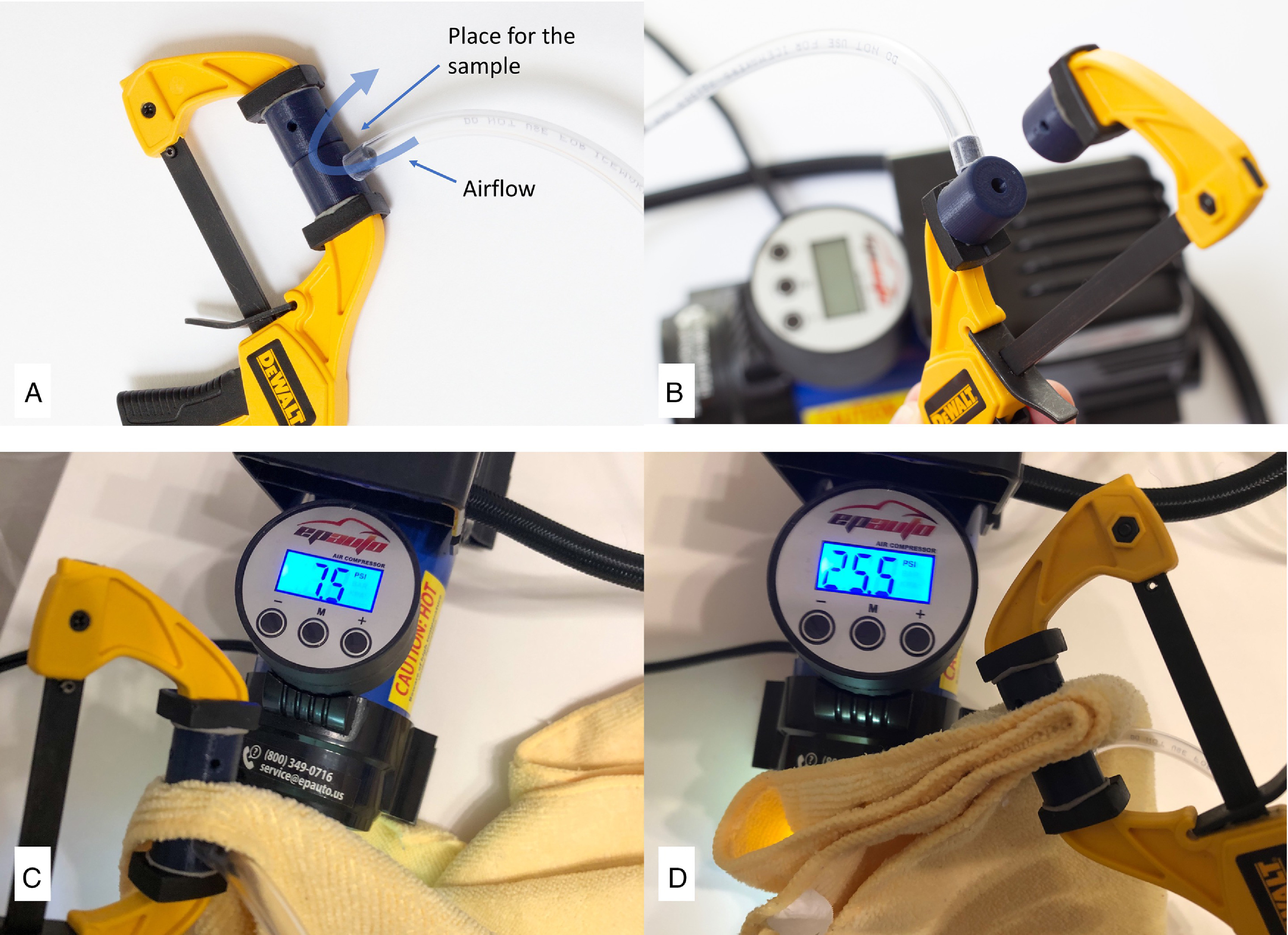
Measurement of Airflow Resistance of a Fabric Sample. A,B, Each sample was compressed between 2 pads (custom 3D printed part), with a 10 mm^2^ size hole in the middle for the air flow, generated by an air compressor. Airflow resistance was 7.5 PSI with a single layer of microfiber cleaning cloth (C) and 25.5 PSI with four layers of the same material (D).

Airflow resistance was measured as pressure drop through the fabric sample using an air compressor with pressure gauge (EPAuto, Model 1. Walnut, CA) (Figure 2). The sample was compressed between 2 pads with a 10-mm^2^ size hole in the middle for the airflow (custom 3D printed part). The lowest measurable resistance (pressure) value was 3.0 psi. Three measurements of each sample were repeated at different locations on the sample. Mean and SEM were calculated.

Filtration performance index of each tested fabric was defined as the area under the fitted curve (AUC) of filtration efficiency versus airflow resistance percentage of the highest N95 mask resistance recorded (15.5 psi).

Various woven fabrics, knit fabrics, nonwoven fabrics and standard mask materials were examined. The term “nonwoven” denotes fabrics that are neither woven, nor knitted. Tested fabrics included microfiber cloth, tea towel, 2 types of T-shirts, hospital scrub, thick fleece, thin fleece, Buff headwear, pillowcase, woven cotton fabric by yard, a scarf, vacuum cleaner bag, multiple types of felt, paper kitchen towel, paper facial tissue, surgical drape, and surgical gown, a surgical mask, and 2 types of N95 masks. Tables 1 and 2 demonstrate composition details and various properties of the tested fabrics. Also, please see Supplemental Material for visual representations of each tested fabric. Up to 3 levels of layering (1, 2, and 4) were tested. Filtration efficiency and airflow resistance across all tested samples were aggregated when examining the differences due to layering. Analyses including nonlinear regression and Kruskal-Wallis nonparametric test with post-hoc Dunn’s multiple comparisons were performed using Prism (GraphPad, Version 7.00. San Diego, CA). A 2-tailed *P*-value < 0.05 was considered significant.

**TABLE 1:** Airflow Resistance and Filtration Efficiency of Woven and Knitted Fabrics

Material	Type	GSM/layer (g/m^2^)	Layers	Mean Resistance (PSI)	SEM Resistance (PSI)	Mean Filtration Efficiency	SEM Filtration Efficiency
Microfiber cloth - 80% polyester - 20% polyamide, TPI: 38	Woven	300	4	24	1.04	99.66%	0.02%
			2	14.67	0.33	94.43%	0.10%
			1	7.33	0.17	71.30%	0.18%
Tea towel - 100% cotton, TPI: 38	Woven	388	2	7.67	0.17	88.18%	1.10%
			1	4.5	0	72.13%	2.61%
T-shirt - 60% cotton 40% polyester	Knitted	161	4	7.33	0.17	86.41%	0.11%
			2	3.83	0.17	68.39%	3.31%
			1	*	*	60.63%	0.39%
Hospital scrubs - 55% cotton 45% polyester, TPI: 160	Woven	160	2	8.67	0.67	85.51%	0.10%
			1	5.5	0.29	76.70%	1.99%
T-shirt - 100% cotton	Knitted	204	4	10.67	0.33	84.68%	0.18%
			2	5.67	0.17	68.20%	0.56%
			1	3	0	41.04%	0.49%
Thick fleece - 100% polyester	Knitted	218	2	4.5	0	80.13%	1.04%
			1	*	*	65.77%	0.56%
Thin fleece - 100% polyester	Knitted	167	2	4.17	0.17	63.12%	0.69%
			1	*	*	48.77%	0.81%
Thin, non-elastic woven fabric - 100% cotton, TPI: 128	Woven	151	4	11.33	0.33	61.33%	0.28%
			2	7.5	0	43.89%	0.48%
			1	4.33	0.17	33.44%	0.85%
Buff headwear - 100% polyester	Knitted	139	2	3.17	0.17	58.92%	1.72%
			1	*	*	37.94%	1.44%
Pillowcase - 100% cotton, TPI: 210	Woven	130	2	10.17	0.17	50.57%	0.41%
			1	6.33	0.44	39.87%	0.71%
Scarf - 100% silk, TPI: 270	Woven	22	2	3	0	41.69%	0.87%
			1	*	*	27.64%	0.72%

Abbreviations: TPI, threads per square inch; GSM, gram per square meter; SEM, standard error of mean.

*Resistance below 3 psi was not recorded.

**TABLE 2:** Airflow Resistance and Filtration Efficiency of Nonwoven Fabrics

Material	GSM/Layer (g/m^2^)	Layers	Mean Resistance (PSI)	SEM Resistance (psi)	Mean Filtration Efficiency	SEM Filtration Efficiency
N95 3M respirator (NIOSH Aura 1870+)		1	5	0	99.99%	0.00%
N95 Halyard respirator (NIOSH TC 84A-7521)		1	15.5	0.76	99.99%	0.00%
Vacuum cleaner bag	125	1	5.17	0.17	98.73%	0.02%
Surgical drape – Spunbond meltblown spunbond (SMS)	37	4	10.17	0.33	98.61%	0.03%
		2	6.5	0	94.15%	0.11%
		1	3.83	0.17	81.30%	1.21%
Surgical mask	58	2	12.67	0.44	96.81%	0.18%
		1	7	0.5	86.40%	0.83%
Felt, 2 mm soft - 100% polyester	189	4	5.33	0.17	96.47%	0.14%
		2	3.83	0.17	89.54%	0.46%
Felt, 1.5 mm soft - 100% polyester (grey)	175	4	4.17	0.17	90.76%	0.10%
		2	*	*	78.79%	0.13%
		1	*	*	60.49%	0.55%
Felt, 1 mm firm - 100% polyester	158	4	3.17	0.17	87.36%	0.13%
		2	*	*	72.86%	0.26%
		1	*	*	56.04%	1.02%
Paper kitchen towel	66	2	6	0.29	86.07%	2.33%
		1	4	0	76.60%	0.90%
Paper kitchen towel measured a day later		1	4	0.29	55.05%	0.66%
Felt, 1.5 mm soft - 100% polyester (brown)	140	4	3.67	0.17	85.57%	0.16%
		2	*	*	69.76%	0.27%
		1	*	*	54.56%	1.71%
Surgical gown - Spunbond meltblown spunbond (SMS)	42	1	3.83	0.33	79.01%	0.51%
Paper facial tissue	42	2	5.67	0.33	58.64%	0.29%
		1	3.83	0.33	38.73%	1.13%
Nothing - negative control	0	0	*	*	0.84%	0.98%

Abbreviations: GSM, gram per square meter; SEM, standard error of mean.

*Resistance below 3 psi was not recorded.

## RESULTS

Airflow resistance and filtration efficiency results are presented in Table 1 (woven and knit fabrics) and Table 2 (nonwoven fabrics).

Filtration efficiency and airflow resistance differed significantly among varying number of layers (*P* < 0.0001 and *P* < 0.01, respectively). Post-hoc comparisons demonstrated higher filtration efficiency and airflow resistance of 4 layers compared with 2 layers (*P* < 0.001 and *P* < 0.01, respectively) and 1 layer of material (*P* < 0.0001 and *P* < 0.01, respectively), as well as higher filtration efficiency and airflow resistance of 2 layers compared with 1 layer of material (*P* < 0.001 and *P* < 0.05, respectively). There was no significant difference in filtration efficiency and airflow resistance before and after washing the fabric (both *P* > 0.05).

**FIGURE 3:**
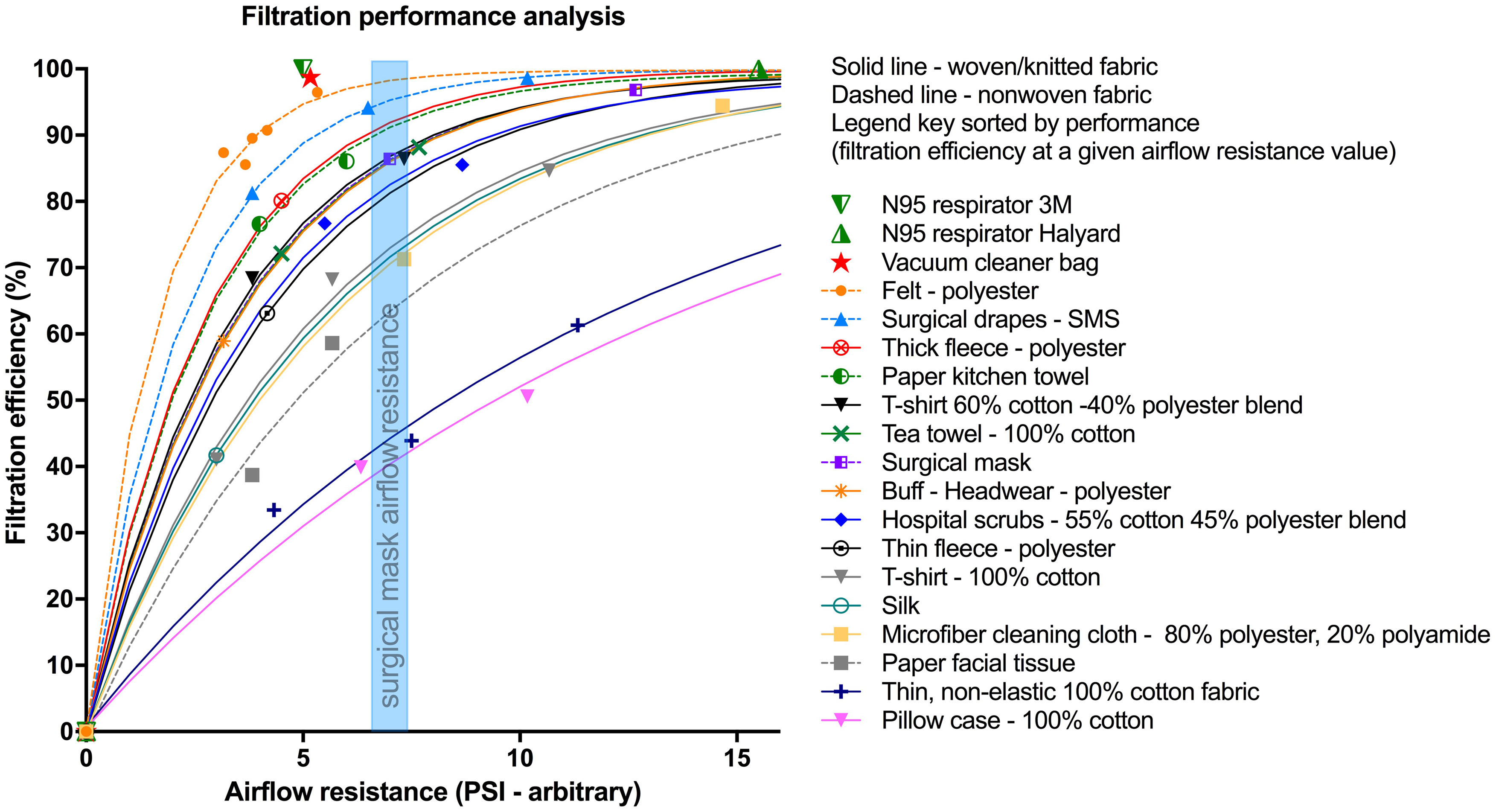
Fabric Filtration Efficiency and Airflow Resistance With Fitted Exponential With 1-Phase Association Filtration Performance Curves. Curves with higher filtration efficiency at a given airflow resistance corresponded to higher filtration performance. Testing results of the N95 respirators and vacuum cleaner bag were plotted as a single data point, respectively. The vertical blue shaded area represents the airflow resistance of a single layer surgical mask, to aid visual comparison of filtration efficiencies of other fabrics.

Fabric performance was plotted and fitted to exponential curves with one phase association (Figure 3). Filtration performance index, as calculated by AUC, was significantly different among the fabrics (*P* < 0.0001). There was a significant downward trending of filtration performance index in the order presented in Figure 3 (*P* < 0.0001) with felt having the highest rank. Given this trend, post-hoc comparisons were performed between felt and all other tested sample groups to further evaluate its filtration performance. Results showed felt with significantly higher filtration performance index (0.86 ± 0.01) compared with cotton-polyester blend T-shirt (0.74 ± 0.02) and hospital scrub (0.70 ± 0.06) (*P* < 0.05 and *P* < 0.05, respectively), 100% cotton materials including tea towel (0.73 ± 0.01), T-shirt (0.65 ± 0.04), thin woven fabric (0.43 ± 0.04), and pillowcase (0.39 ± 0.05) (*P* < 0.05, *P* < 0.05, *P* < 0.001, and *P* < 0.001, respectively), as well as surgical mask (0.70 ± 0.01), microfiber cloth (0.60 ± 0.03), and paper facial tissue (0.57 ± 0.07) (*P* < 0.05, *P* < 0.001, and *P* < 0.001, respectively).

## DISCUSSION

As the COVID-19 pandemic rapidly evolved, public health measures expanded, including guidance on appropriate face coverings in public situations. The guidance suggesting usage of public face coverings did not include standardized material recommendations for noncommercial mask production and that information is not widely represented in available medical literature. Moreover, a lack of commercial N95 and surgical masks available for communities necessitates accepting imperfect solutions for infection prevention. While homemade masks may better serve as a source control measure, established evidence suggests they also confer a certain level of protection to the wearer. Alternative, noninfectious particulate matter filtration applications may include dust or emission mitigation, where the average size of these particles exceeds the 40-nm particles tested.^12^

This study measured filtration efficiency and airflow resistance of fabrics not previously tested in the medical literature.^10^ A fabric filtration performance index was calculated for better comparison among different materials. The effects of layering and washing were also examined.

Commercial and community mask manufacture commonly involves multiple layers of fabric material.^13,14^ This study demonstrated higher filtration efficiency with layering, yet at the cost of higher airflow resistance. A strategy similar to that of a receiver operator curve analysis was used to compare different fabrics, accounting for both properties simultaneously. Fabric material with higher filtration performance across varying airflow resistance would result in a higher area under the curve and greater filtration performance index.

Polyester felt is a commonly available nonwoven fabric, and it demonstrated significantly higher filtration performance compared with more often used homemade mask fabrics.^15^ Felt also performed better than surgical mask material. Felt is washable with similar filtration efficiency and airflow resistance before and after washing, suggesting reusability. On the other end of the performance spectrum, nonelastic 100% cotton fabrics (often used for homemade masks) performed the worst.

One hundred percent cotton tea towel and cotton-polyester blend fabric demonstrated slightly inferior filtration performance compared with felt and may serve as alternative materials for homemade masks. Thick polyester fleece showed high filtration efficiency, although it may be difficult to layer. Microfiber cleaning cloth demonstrated high filtration efficiency, but also high airflow resistance, resulting in a low filtration performance index.

Nonwashable fabrics can be considered for single use if available. Surgical drape/gown material showed high filtration performance. We also tested paper towels for comparison, because communities have broadly considered using it. Of note, paper towels lack consistency across various brands and may tear, especially if wet, reducing filtration reliability.

This study replicated previously demonstrated high filtration efficiency of vacuum cleaner bags^10^ and also showed its desirable low airflow resistance. Although this was not part of the test, we experienced an unpleasant smell while wearing a mask using vacuum cleaner bag material and with other fabrics as well, which may have originated from long-term storage. With washable fabrics, the odor could be eliminated by washing it; therefore, we recommend washing masks before wearing.

Intrinsic fabric filtration characteristics between woven, knit, and nonwoven fabrics vary due to manufacturing differences. For woven or knitted fabrics, the fibers are made into yarn first, then those yarns are woven or knitted into fabrics. Woven fabrics are manufactured by the interlacement of fibers in an organized manner, yielding a uniform, essentially 2-dimensional structure. Knitted fabrics are produced by interloping the yarn. Alternatively, so-called nonwoven fabrics are bonded together by entangling fibers or filaments by means of various mechanical, thermal, and chemical processes, resulting in a 3-dimensional configuration. Nonwoven fabrics are used as filters and widely considered superior in filtration performance to their woven and knit counterparts. High airflow resistance of woven fabrics make them suitable for windbreakers, because the yarn itself causes resistance, and if tightly woven, the air passing through the pores can be limited. Knitted fabrics in general are looser and more elastic, compared with fabrics that are woven. Nonwoven fabrics do not contain high resistance yarn. The thin, randomly oriented fibers result in less resistance, small pore size, and larger active surface area, which increase the probability of particles becoming trapped, thus increasing filtration performance^16,17^ The results of this study reflect those differences, with nonwoven materials outperforming woven and knitted fabrics.

Among other factors, material composition may also play an important role in filtration efficiency due to the surface charge of the fibers contributing to varying degrees of traversing particle electrostatic capture.

In general, we believe that higher performing material is beneficial in both infectious source control and personal protection for the wearer. Filtration performance of fabrics includes the filtration efficiency (the higher the better) and airflow resistance (the lower the better). For mask design, the filtration efficiency shows the fraction of filtered particles if the air passes through the fabric; however, if the airflow resistance is high, the air will more likely find other ways and bypass the fabric unfiltered when entering or exiting the mask, thus decreasing the actual filtration efficiency.

This study has some limitations. First, airflow resistance values less than 3 psi were not recorded given equipment limitations. Second, only viral sized particles were tested; however, earlier publications examined viral sized and bacterial sized particles. They found that, for most materials, the filtration efficiency of a given fabric was generally higher for larger particles than for smaller, viral sized particles.^10^ Third, this study tested the material only and not actual masks. Potential properties of a given fabric may be negated or complemented by mask design and overall facial fit, which will be significant variables in communities independently producing countless variations of nonstandardized designs. Indeed, prior studies have demonstrated the importance of proper fit on both source control and receiver protection.^18^ Additionally, increasing filtration surface area may counter the innate high airflow resistance of a fabric. Different fabric materials can also be combined, including stiffer materials for shaping; malleable materials for better seal; and comfortable materials for skin protection. Also, intrinsic performance indices may vary across different airflow velocities, which are expected across different users’ breathing efforts. These factors and techniques were not examined, but are relevant topics for future investigations.

## CONCLUSIONS

This study has assessed airflow resistance and filtration efficiency of available household and health-care fabrics in varying number of layers and pre-/postwashing states. Filtration performance data of tested fabrics can help maximize effectiveness and comfort of homemade masks, with the goal of decreasing viral transmission during a pandemic where supply of personal protective equipment is limited.

## Supplementary material

For supplementary material accompanying this paper visit https://doi.org/10.1017/dmp.2020.262. click here to view supplementary material
