# Supplementary material for: Material Suitability Testing for Nonmedical Grade Community Face Masks to Decrease Viral Transmission During a Pandemic
Source: Disaster Med Public Health Prep. 2020 Jul 27:1–7. doi: 10.1017/dmp.2020.262 (PMC7450244; doi:10.1017/dmp.2020.262)

Material Suitability Testing for Non-Medical Grade Community Face Masks to Decrease Viral Transmission During a Pandemic

Supplementary material

Csanad Varallyay MD, PhD, Ningcheng (Peter) Li MD,

Brendan Case MD, Bryan Wolf MD

Woven/knitted fabrics

Natural fabrics

Thin, non-elastic fabric - 100% cotton

Layers	airflow resistance	Filtration efficiency (%)
1	4.33	33.44
2	7.5	43.89
4	11.33	61.33

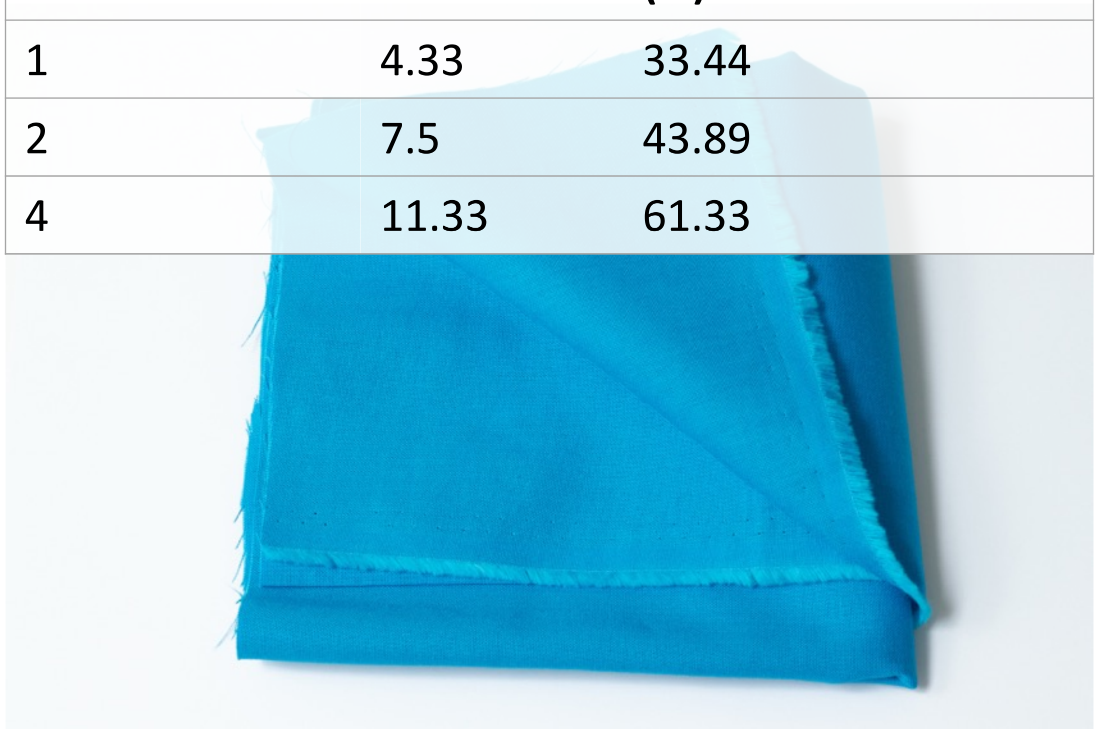

Thin, medium tightly woven, non-elastic fabric.
GSM: 151 g/m^2 TPI:128

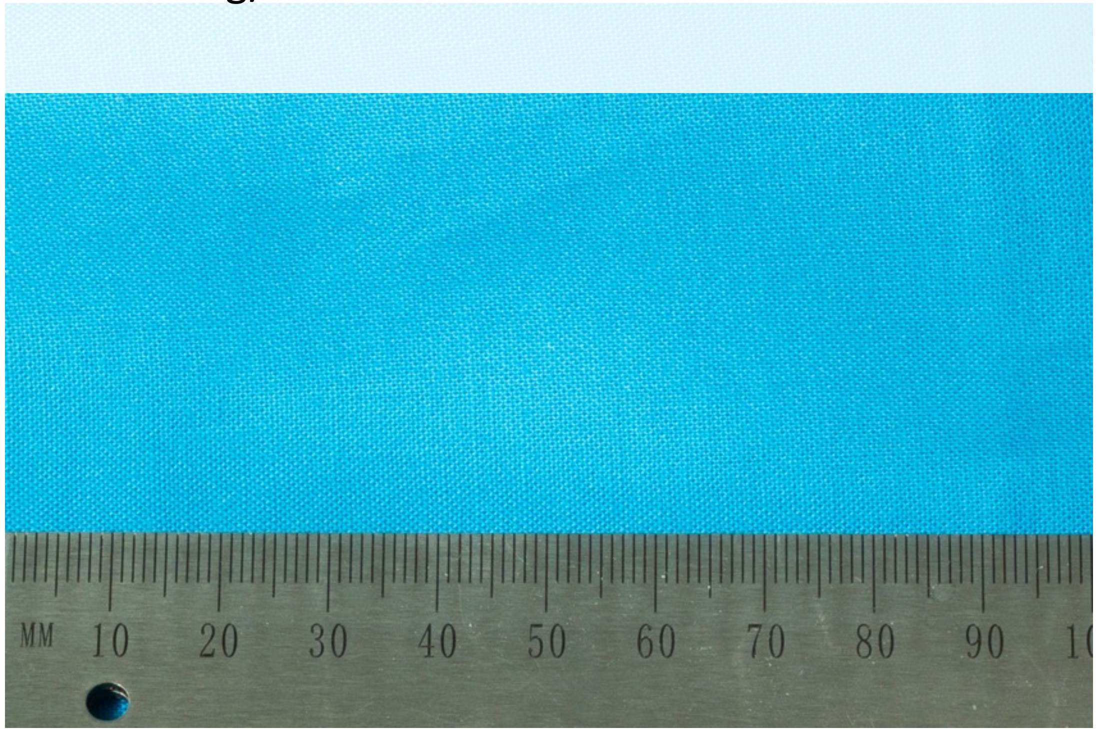

GSM: grams per square meter, TPI: threads per square inch

T-shirt - 100% cotton

Layers	airflow resistance	Filtration efficiency (%)
1	3	41.04
2	5.67	68.2
4	10.67	84.68

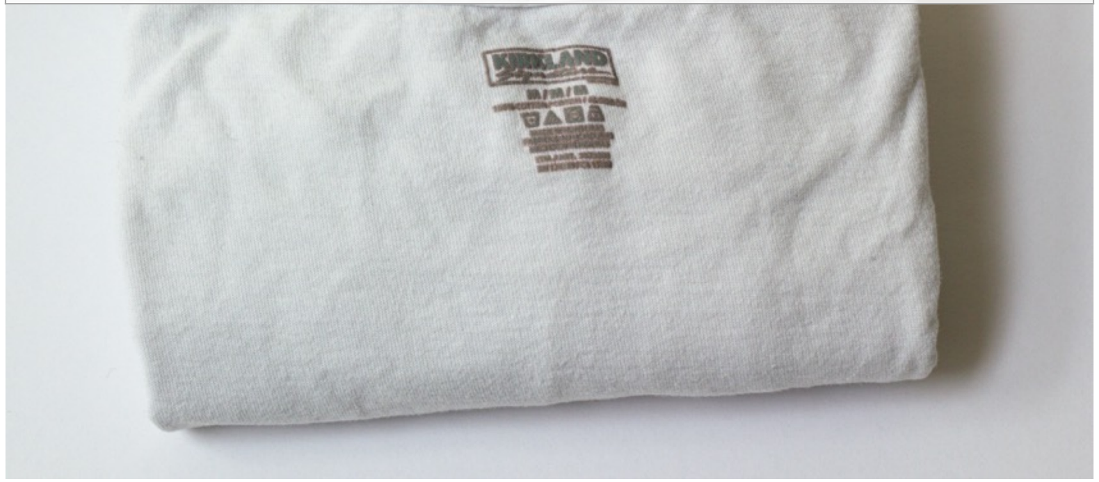

“Usual” thicker 100% cotton knitted T-shirt fabric, mildly elastic. GSM: 204 g/m^2

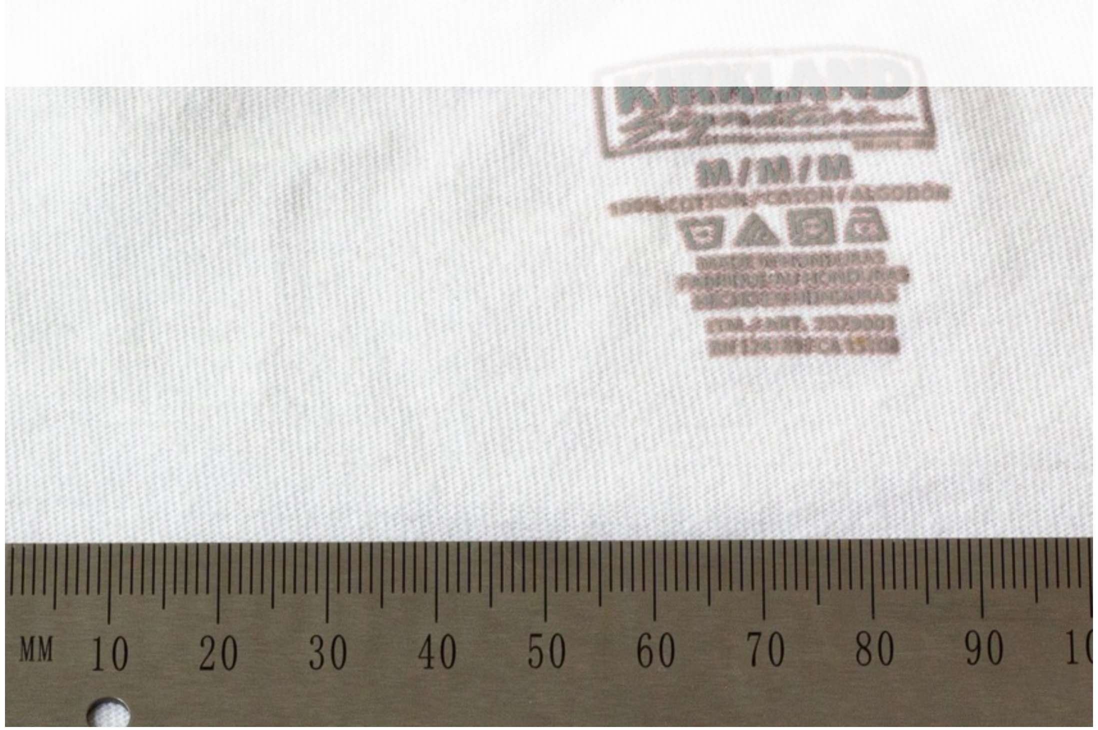

Pillowcase – 100% cotton

Layers	airflow resistance	Filtration efficiency (%)
1	6.33	39.87
2	10.17	50.57

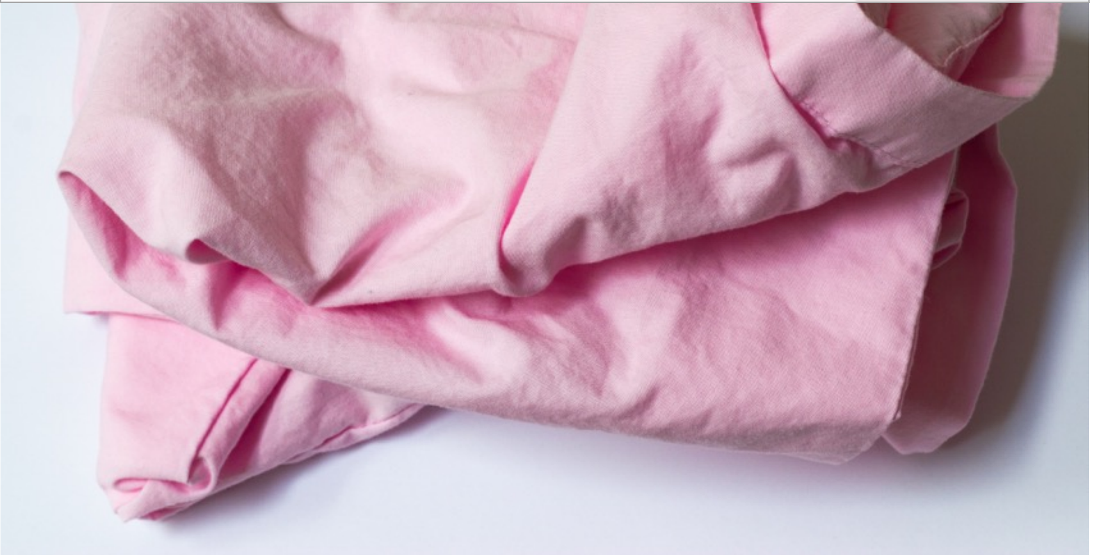

Thin, tightly woven, non-elastic fabric.
GSM: 130 g/m^2, TPI: 210

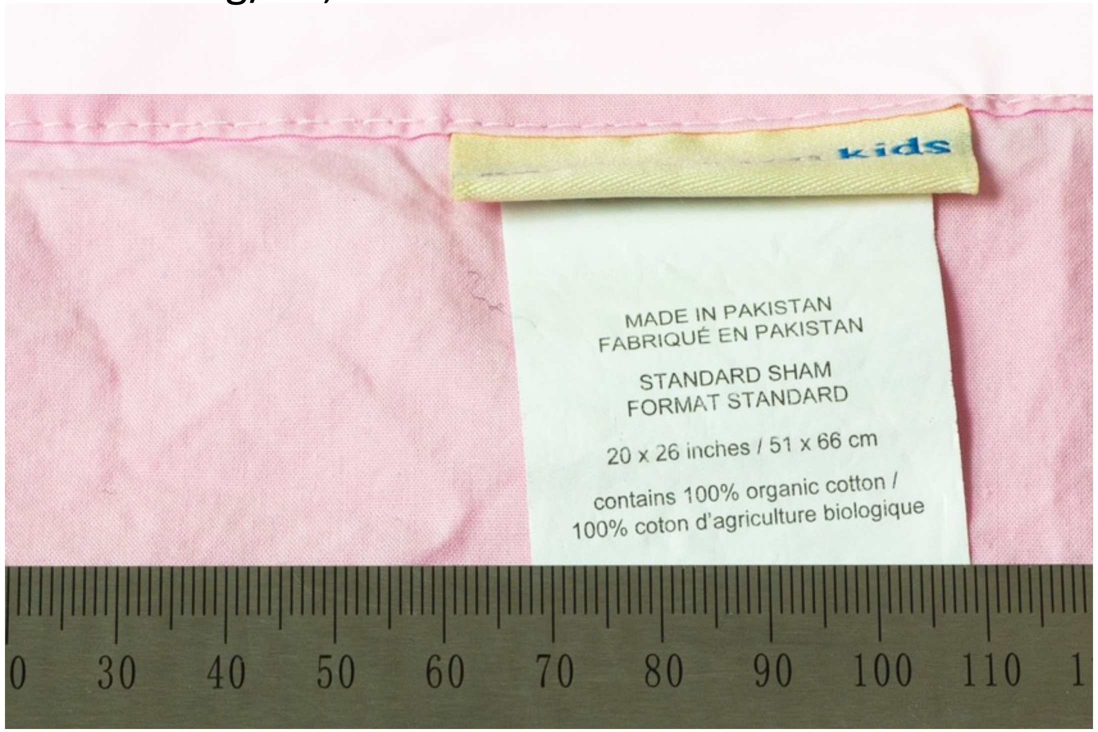

Tea towel – 100% cotton

Layers	airflow resistance	Filtration efficiency (%)
1	4.5	72.13
2	7.67	88.18

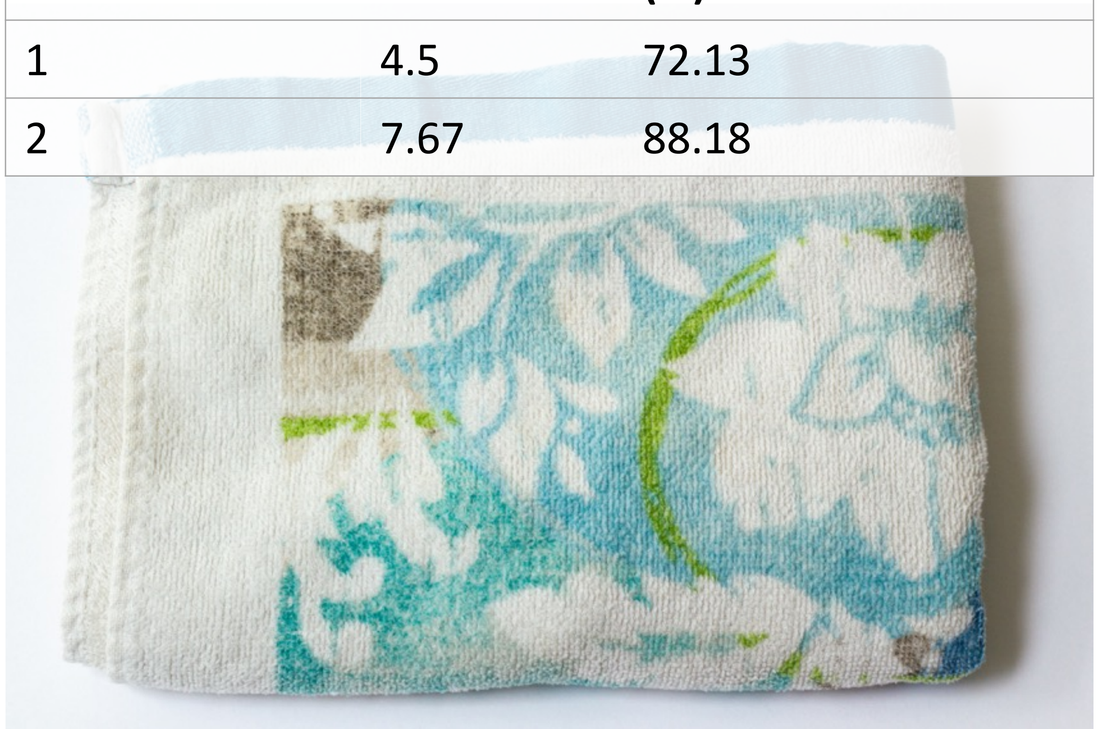

Very thick, soft, 100% cotton woven fabric.
GSM: 388 g/m^2, TPI: 38

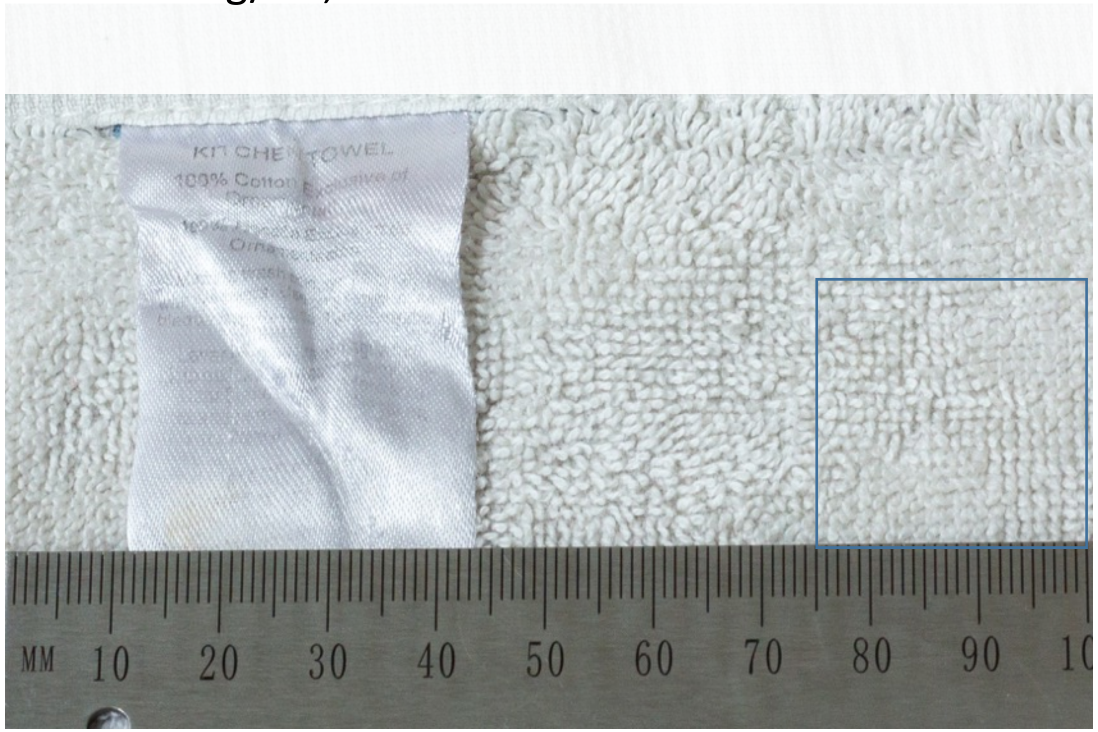

Scarf – 100% silk

Layers	airflow resistance	Filtration efficiency (%)
1	*	27.64
2	3	41.69

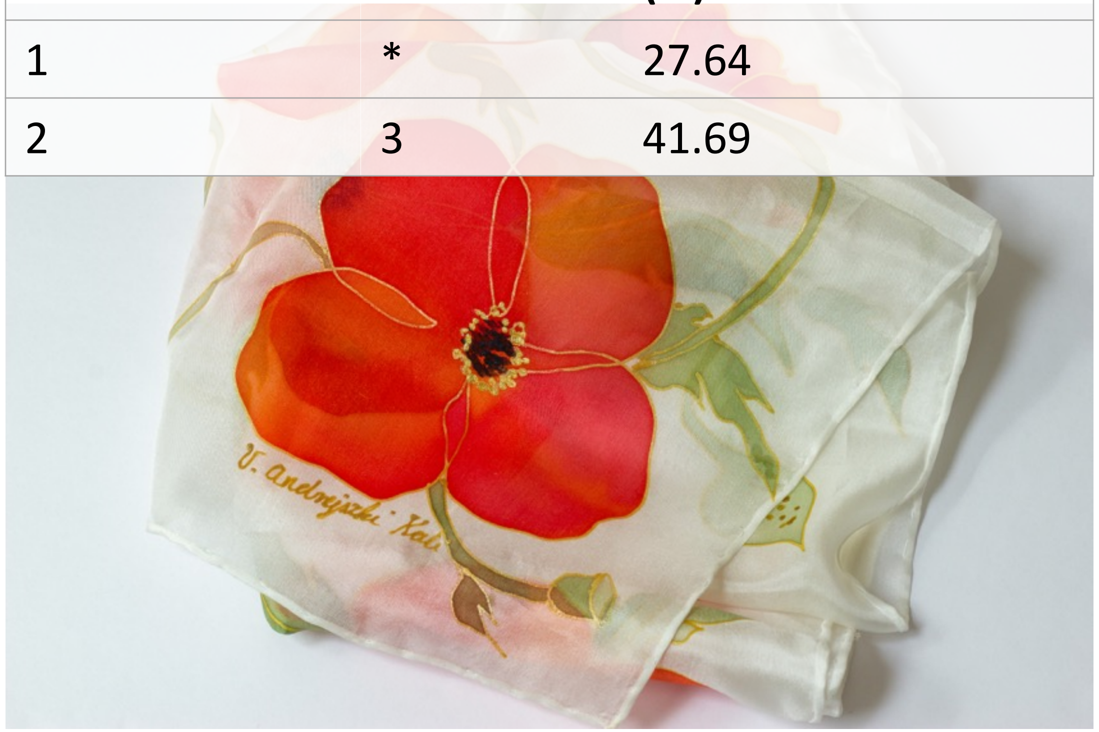

Very thin, woven silk – clear areas were tested.
GSM: 22 g/m^2, TPI: 270

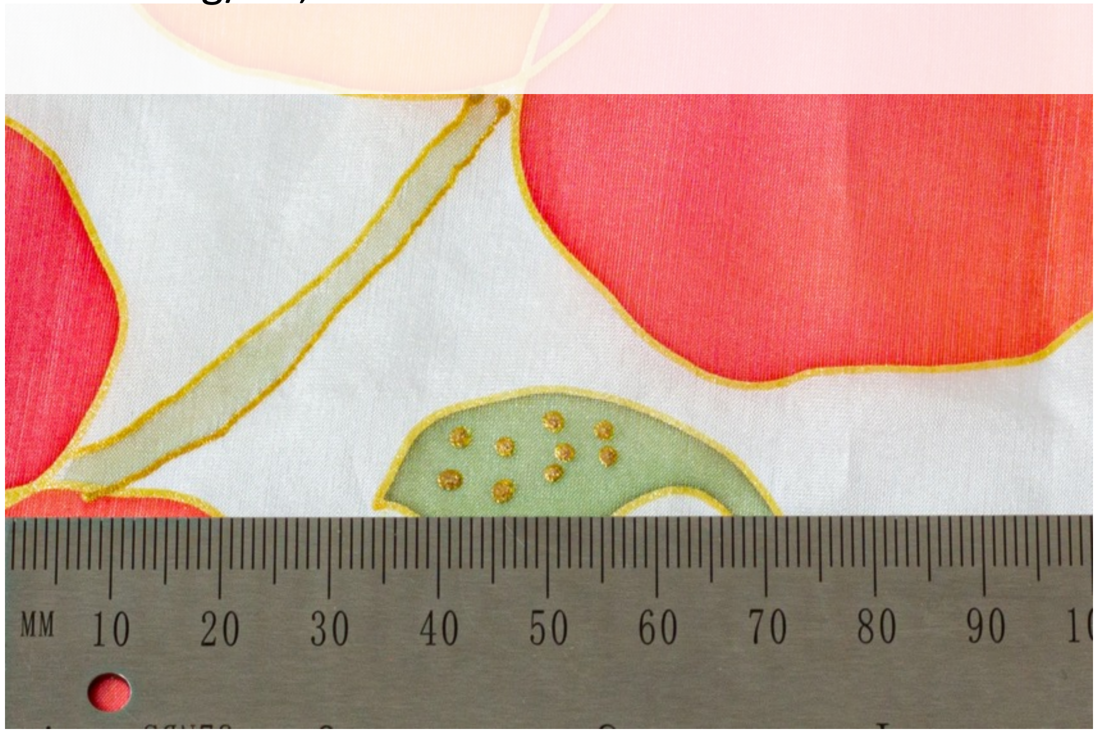

* Value < 3

Woven/knitted fabrics

Natural – synthetic blend

T-shirt - 60% cotton – 40% polyester blend

Layers	airflow resistance	Filtration efficiency (%)
1	*	60.63
2	3.83	68.39
4	7.33	86.41

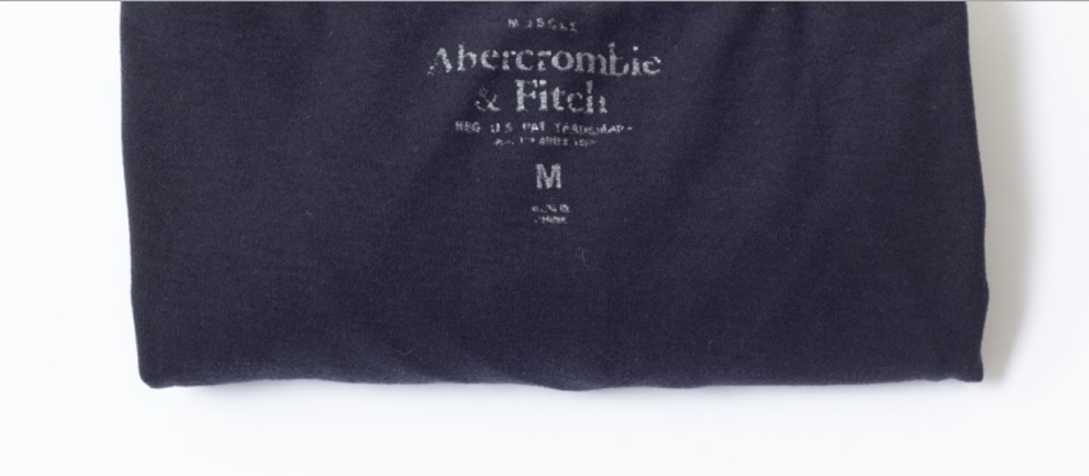

Medium thick, knitted T-shirt fabric, mildly elastic.
GSM: 161 g/m^2

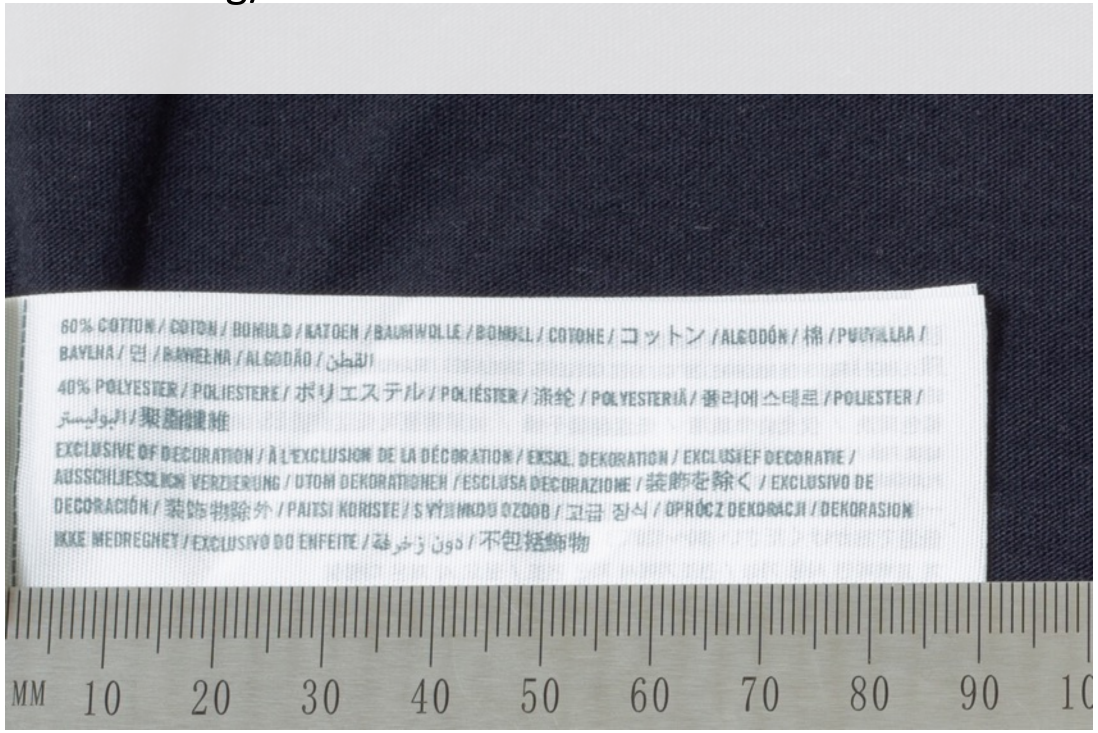

* Value < 3

Hospital scrubs - 55% cotton, 45% polyester blend

Layers	airflow resistance	Filtration efficiency (%)
1	5.5	76.7
2	8.67	85.51

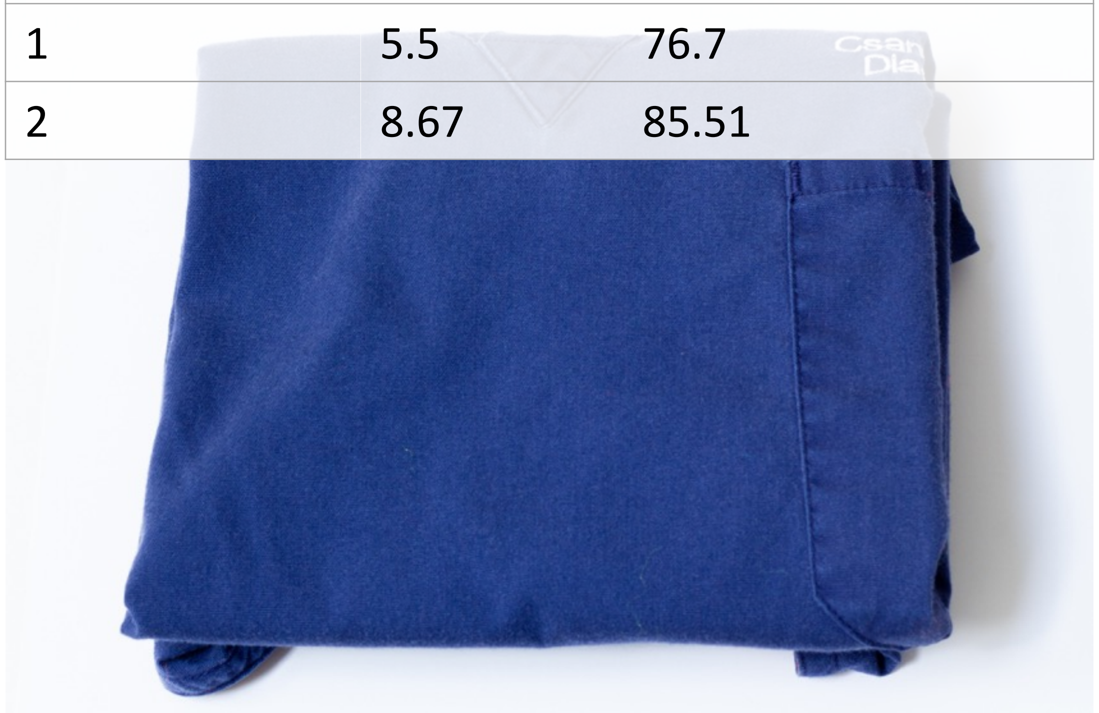

Medium thickness, densely woven, non-elastic fabric.
GSM: 160 g/m^2, TPI: 160

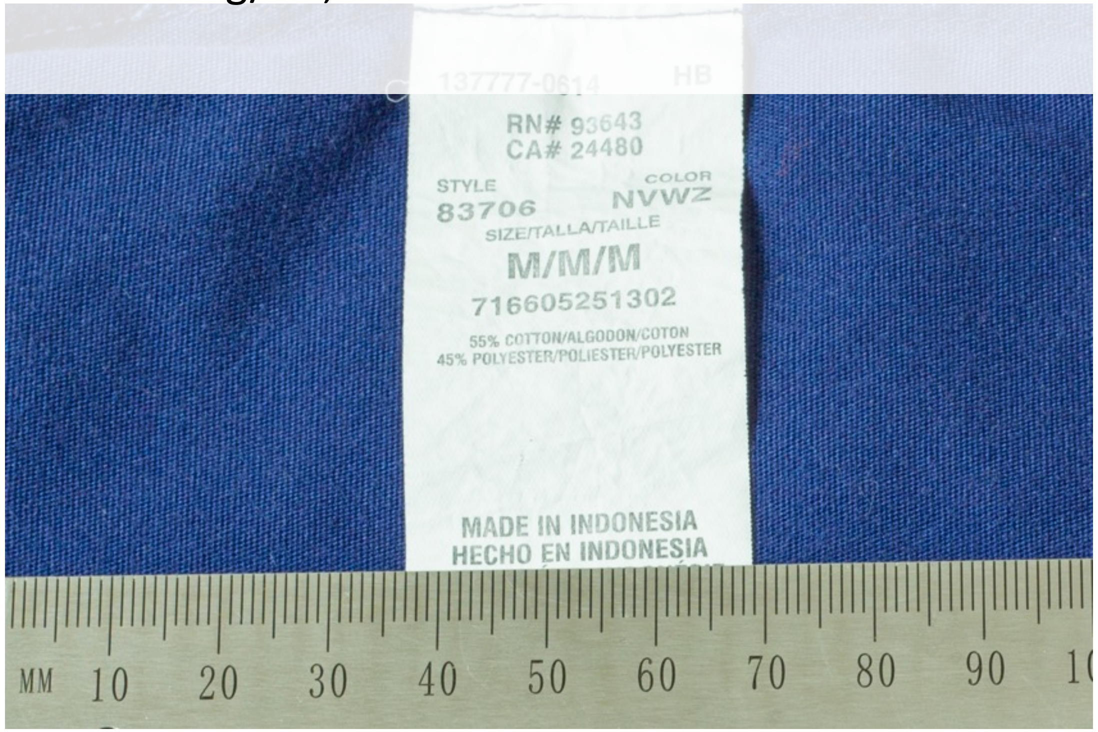

Woven/knitted fabrics

Syntethic

Buff Headwear – 100% polyester

Layers	airflow resistance	Filtration efficiency (%)
1	*	37.94
2	3.17	58.92

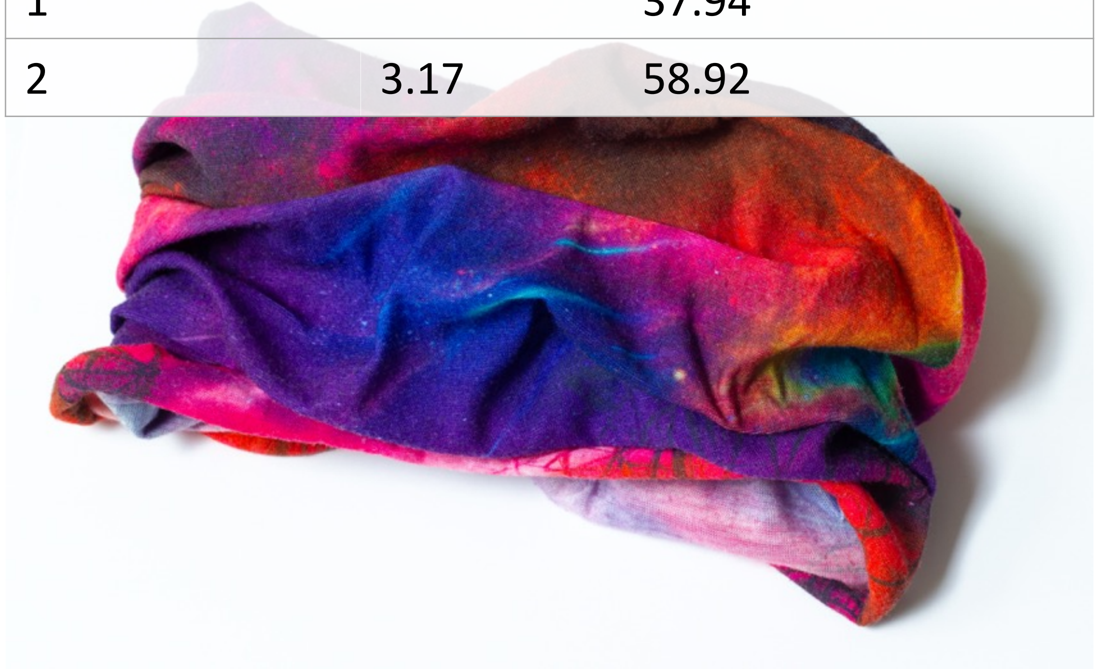

Very elastic, thin, knitted fabric, filtration efficiency and airflow resistance values likely decrease when stretched. GSM: 139 g/m^2

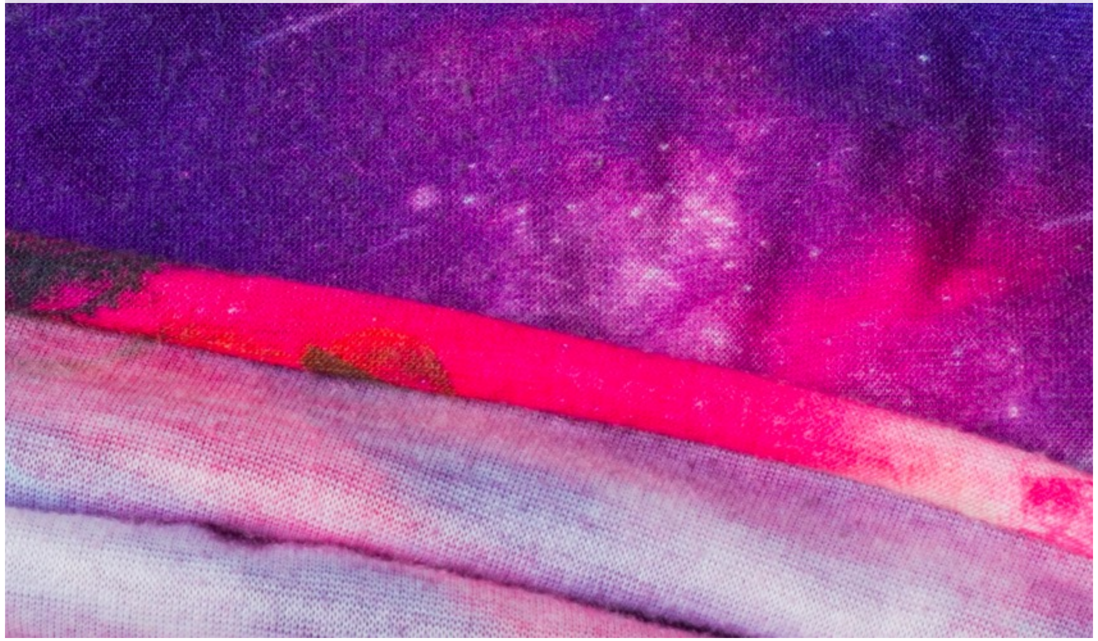

* Value < 3

Fleece sweater (thin) – 100% polyester

Layers	airflow resistance	Filtration efficiency (%)
1	*	48.77
2	4.17	63.12

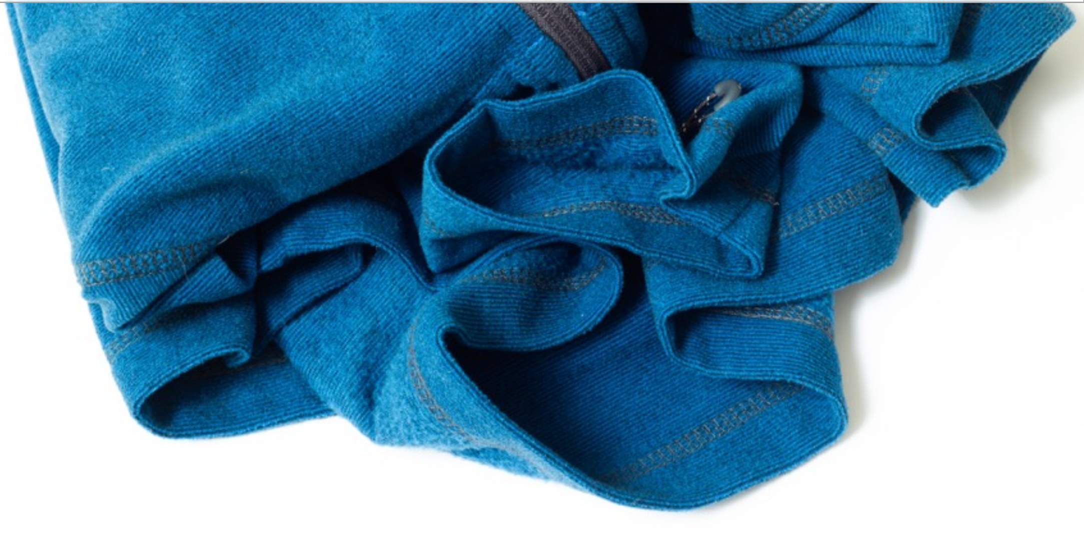

Medium thick, soft, elastic knitted fabric.
GSM: 167 g/m²

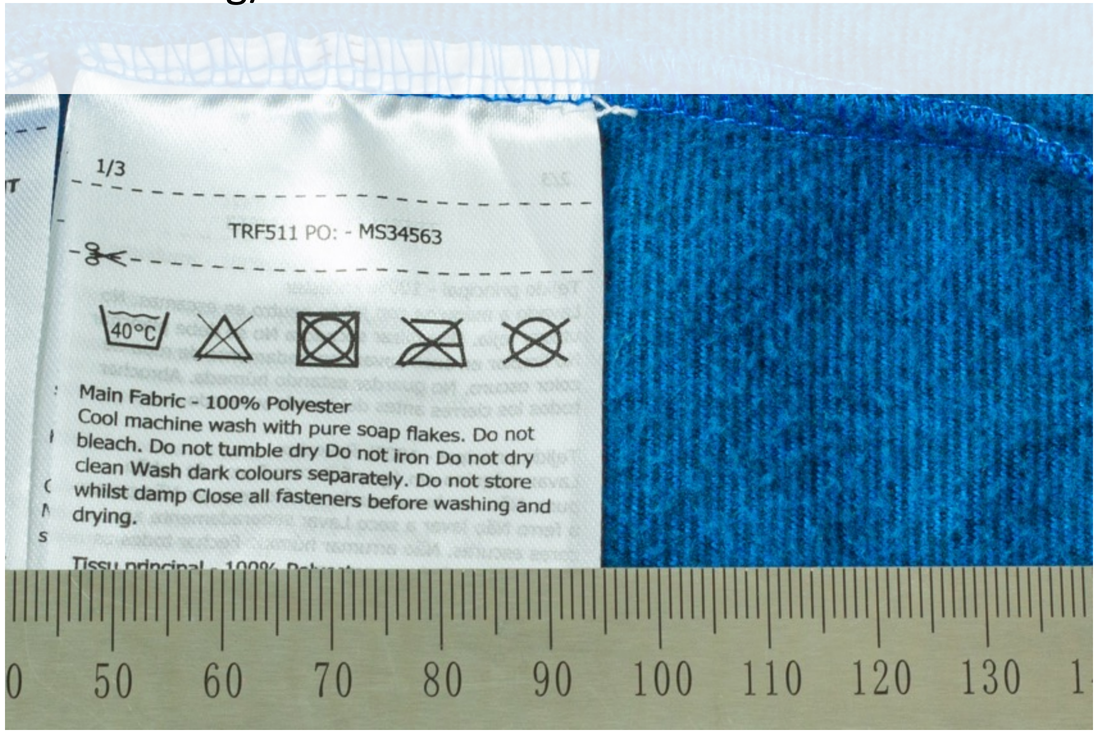

* Value < 3

Fleece sweater (thicker) – 100% polyester

Layers	airflow resistance	Filtration efficiency (%)
1	*	65.77
2	4.5	80.13

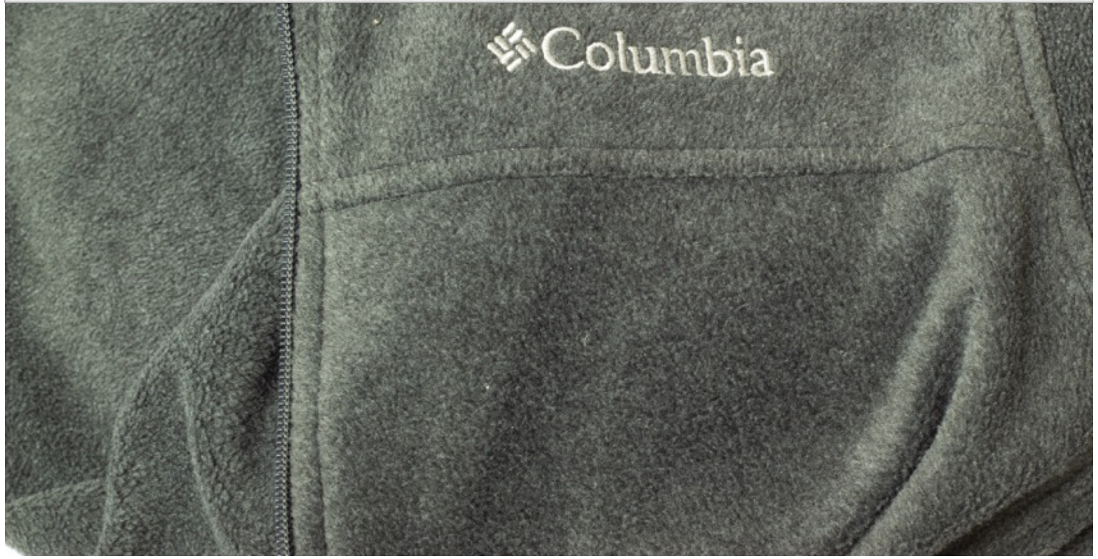

Thick, soft, elastic knitted fabric.
GSM: 218 g/m^2

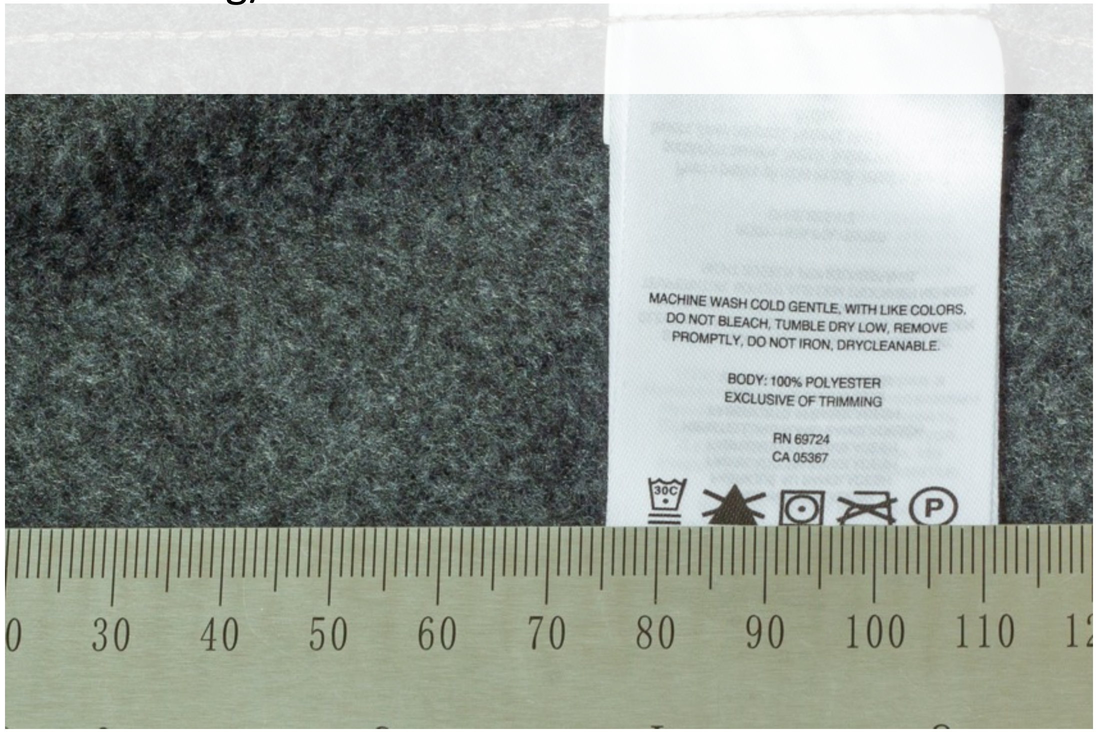

* Value < 3

Microfiber cleaning cloth - 80% polyester, 20% polyamide

Layers	airflow resistance	Filtration efficiency (%)
1	7.33	71.3
2	14.67	94.43
4	24	99.66

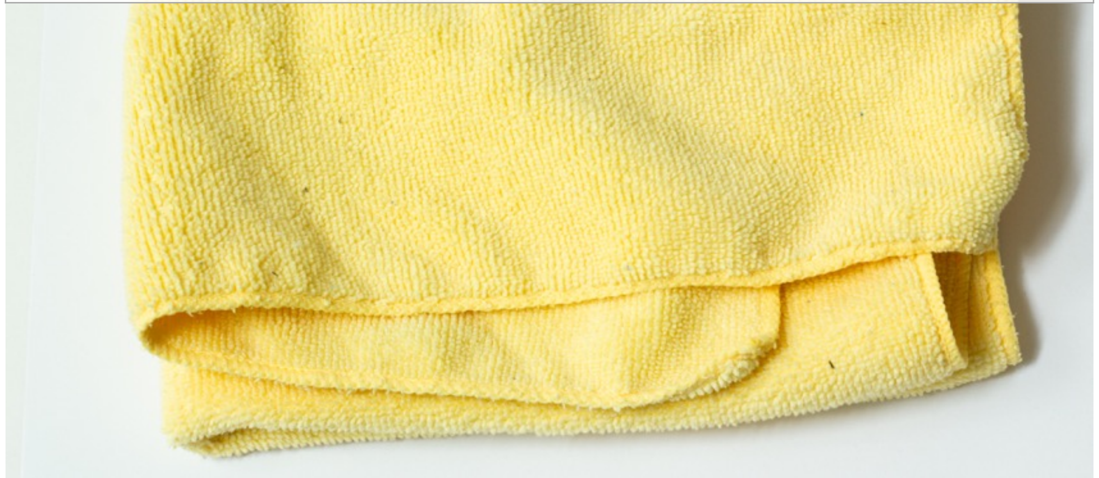

Very thick, very soft, woven fabric.
GSM: 300 g/m^2, TPI: 38

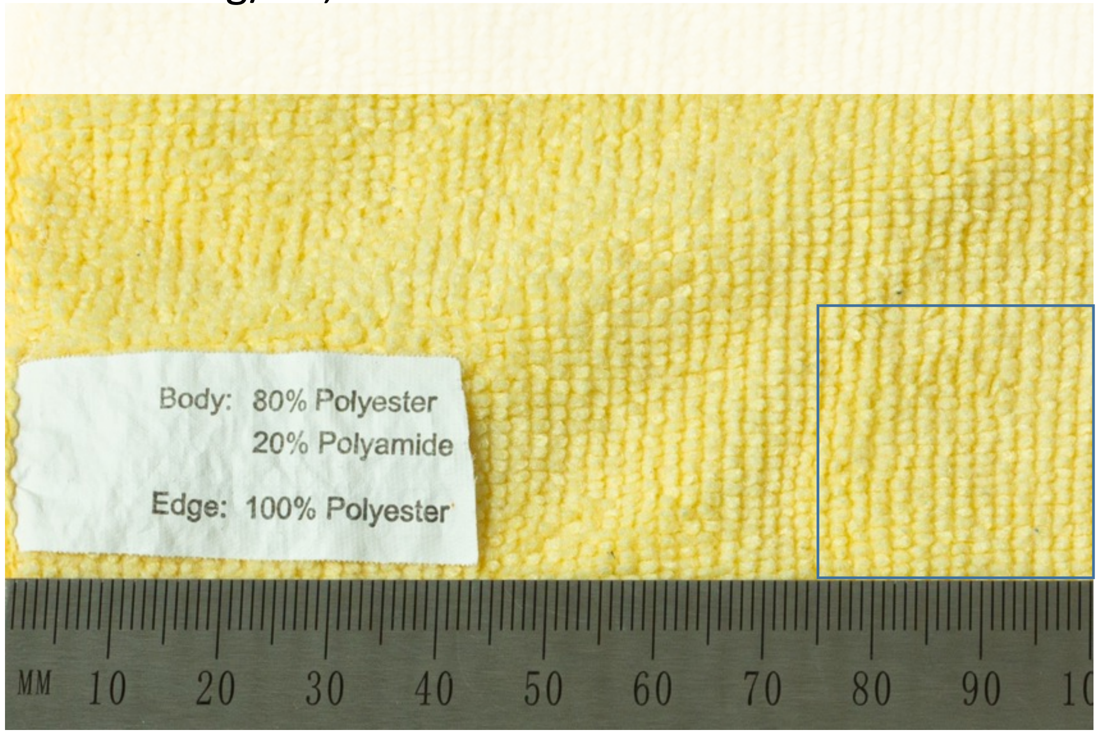

Non-Woven fabrics

Felt (~2mm thick/layer, soft) – polyester

Layers	airflow resistance	Filtration efficiency (%)
2	3.83	89.54
4	5.33	96.47

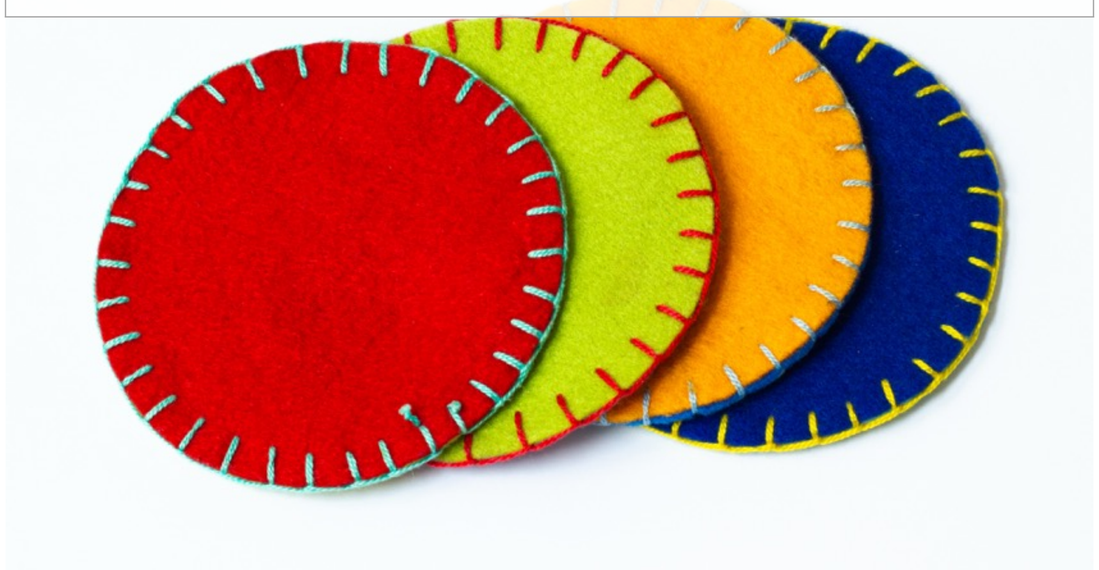

Soft, non-elastic, mildly formable, non-woven fabric.
GSM: 189 g/m^2

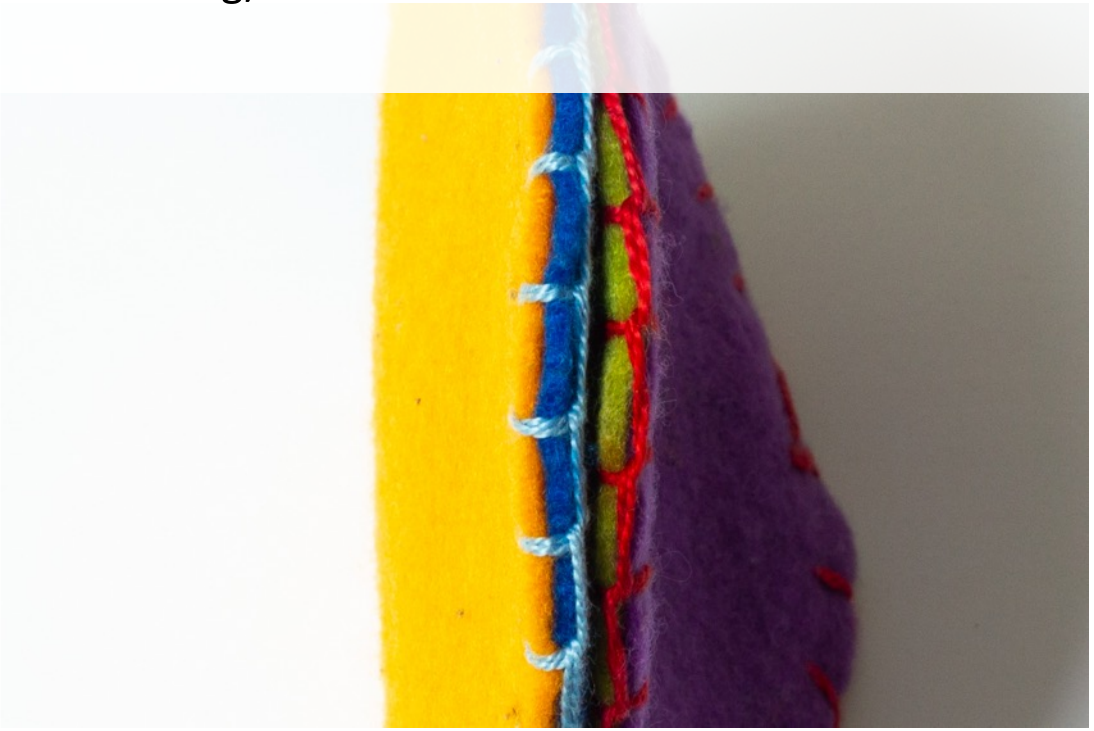

Felt sheet, (soft, 1.5mm thickness) – polyester

Layers	airflow resistance	Filtration efficiency (%)
1 (unwashed)	*	54.56
2 (unwashed)	*	69.76
4 (unwashed)	3.67	85.57
1 (washed)	*	54.95
2 (washed)	*	68.76
4 (washed)	3.5	87.25

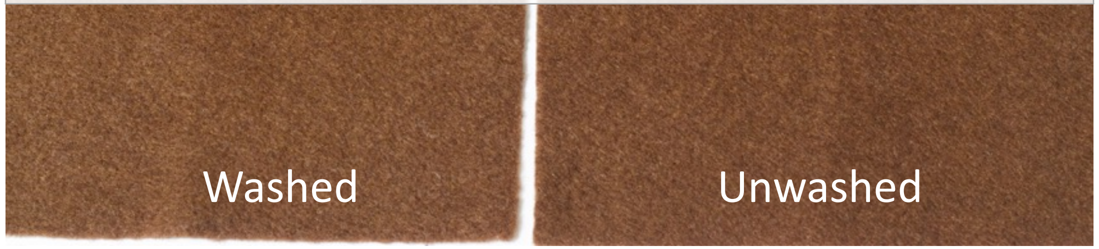

Soft, non-elastic, mildly formable, non-woven fabric.
GSM: 140 g/m^2

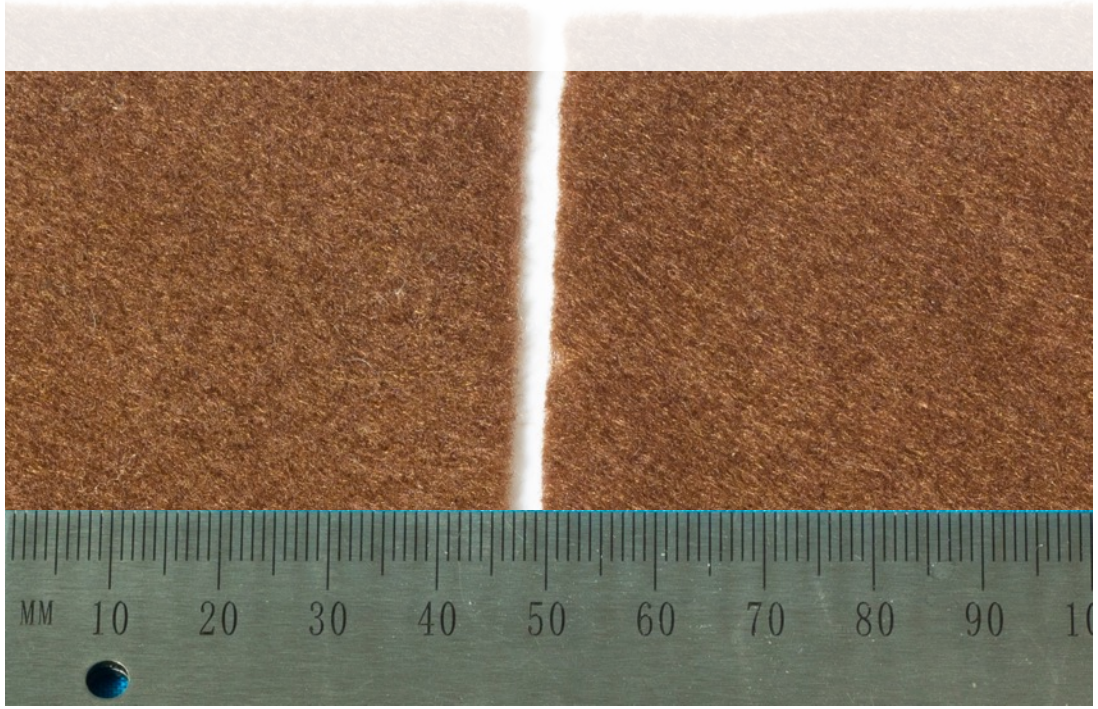

* Value < 3

Felt sheet, (soft, 1.5mm thickness) – polyester

Layers	Resistance	Filtration efficacy (%)
1 (unwashed)	*	60.49
2 (unwashed)	*	78.79
4 (unwashed)	4.17	90.76
1 (washed**)	*	72.13
2 (washed**)	*	89.2
4 (washed**)	4.33	96.39

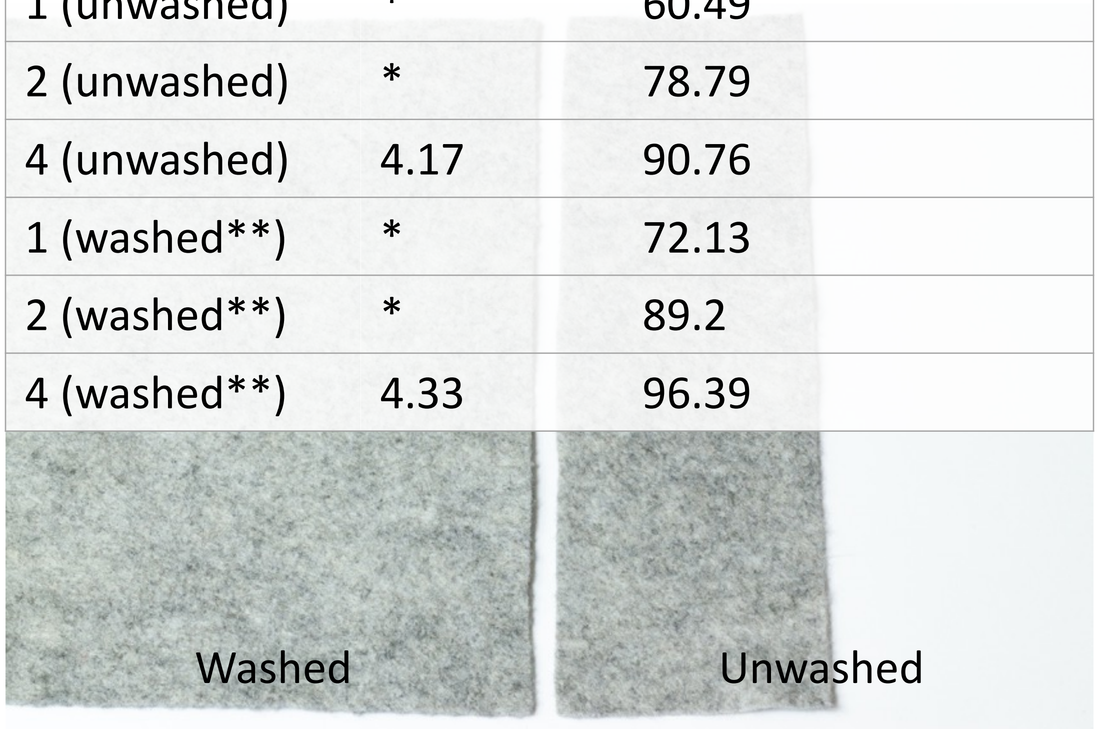

Soft, non-elastic, mildly formable, non-woven fabric.
GSM: 175 g/m[2]

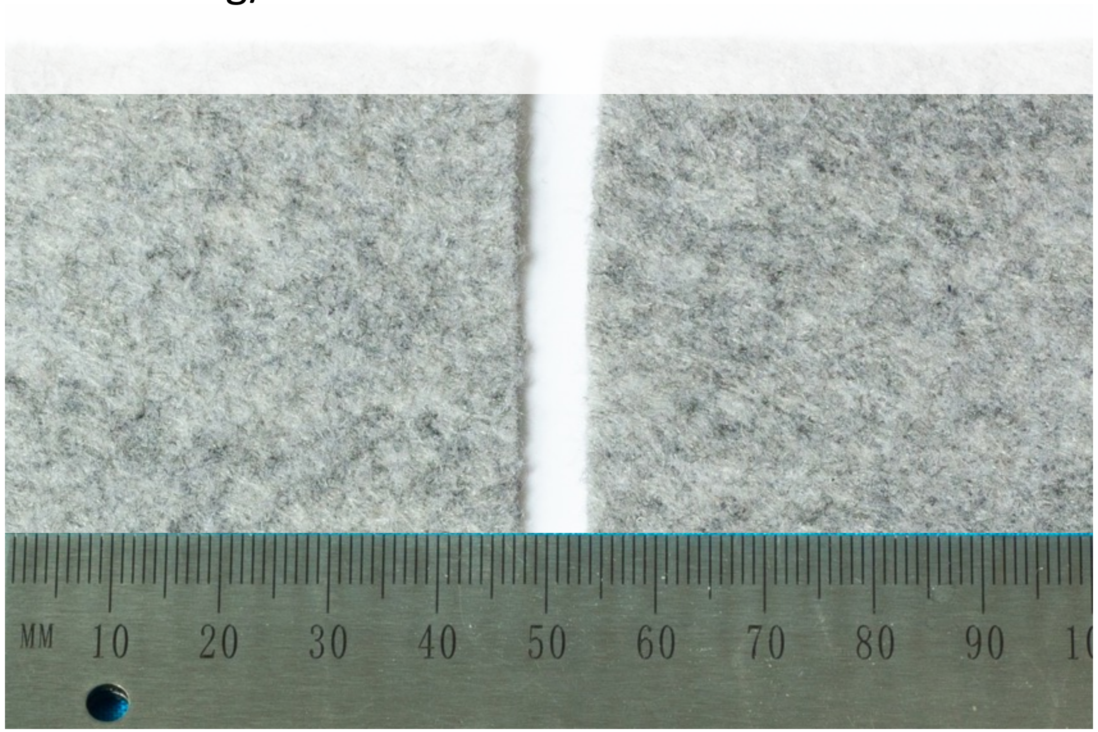

* Value < 3

** Note that this sample remained in the dryer for an extended time, and was taken out shortly before testing. Improvement of filtration efficacy after washing/drying may be secondary to increased electrostatic charge.

Felt sheet, (hard, 1mm thickness) – polyester

Layers	airflow resistance	Filtration efficiency (%)
1 (unwashed)	*	56.04
2 (unwashed)	*	72.86
4 (unwashed)	3.17	87.36
1 (washed*)	*	54.32
2 (washed*)	*	75.29
4 (washed*)	3.67	88.82

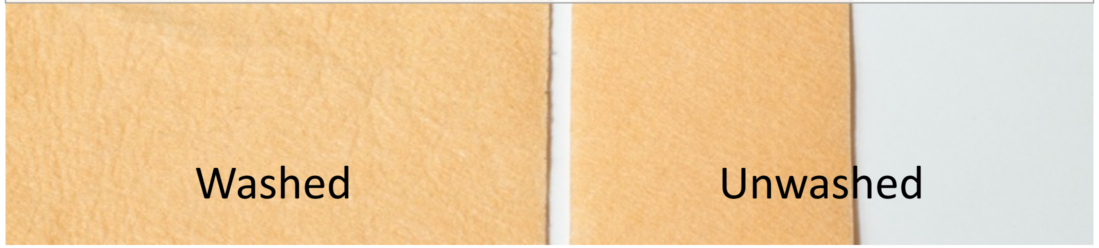

Firm, non-elastic, non-woven fabric.
GSM: 158 g/m^2

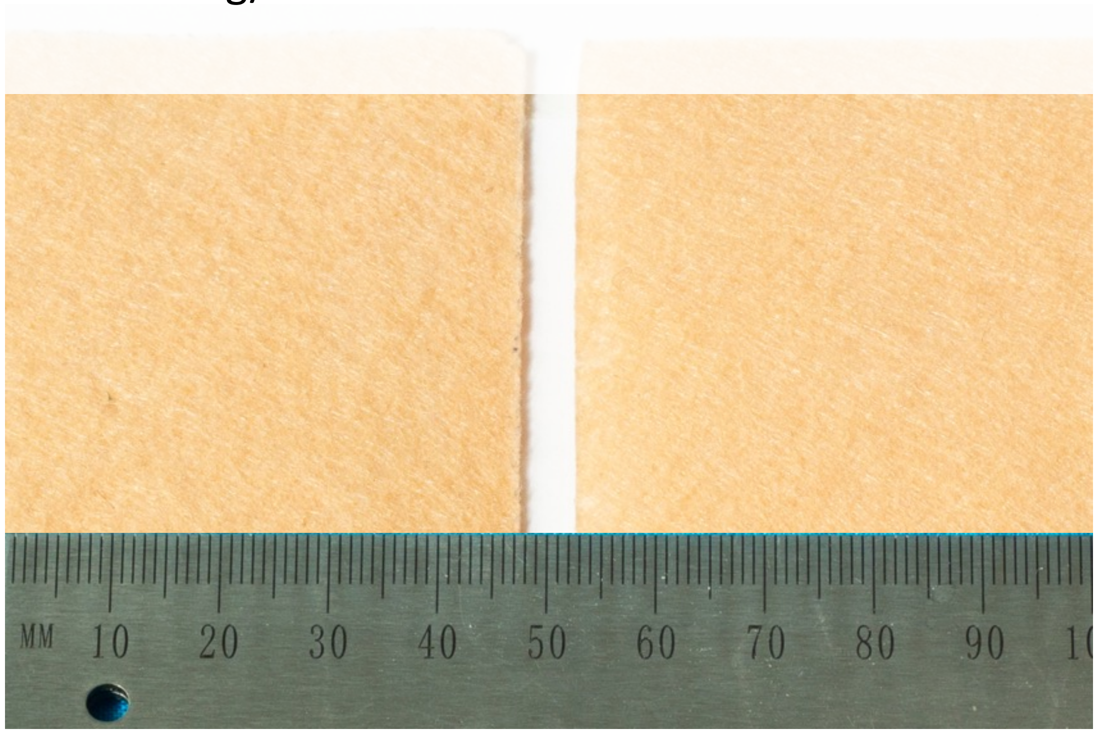

* Value < 3

Thick paper kitchen towel

Layers	airflow resistance	Filtration efficiency (%)
1	4	76.7
2	6	86.07
1 repeat *	4	55.05

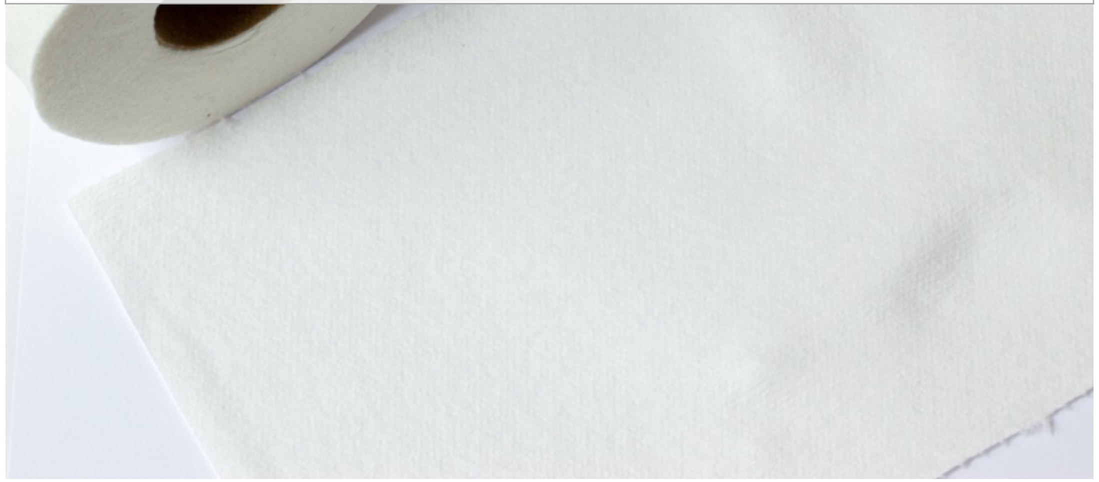

GSM: 66 g/m^2
Initial measurement slowed high filtration, repeated measurement (*) showed lower filtration efficiency. Paper towels may be less consistent in efficiency.

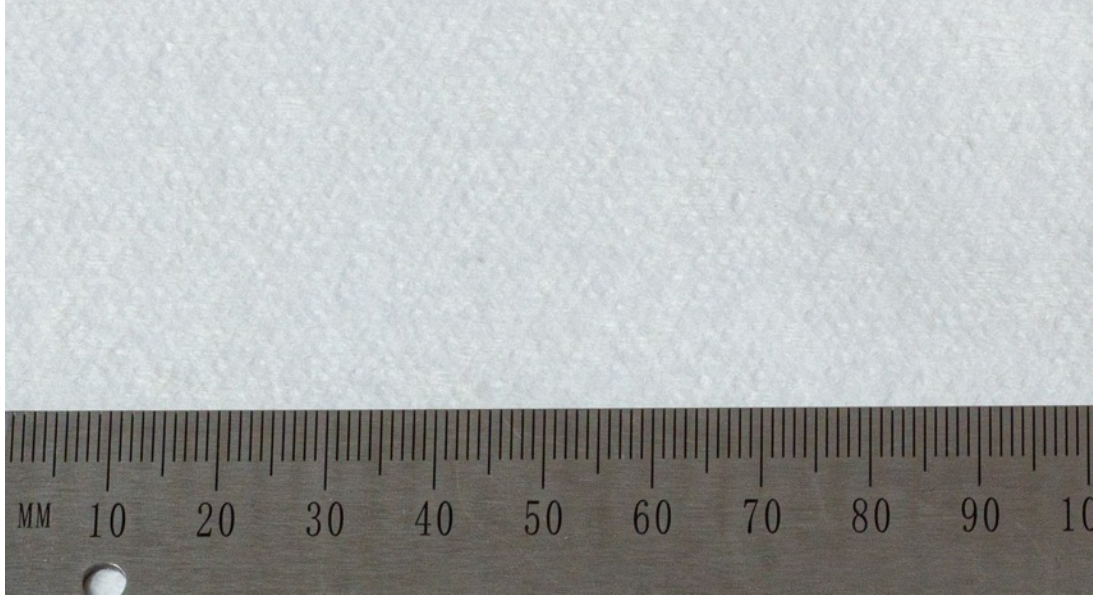

* A different piece was tested a few days later

Paper facial tissue

Layers	airflow resistance	Filtration efficiency (%)
1	3.83	38.73
2	5.67	58.64

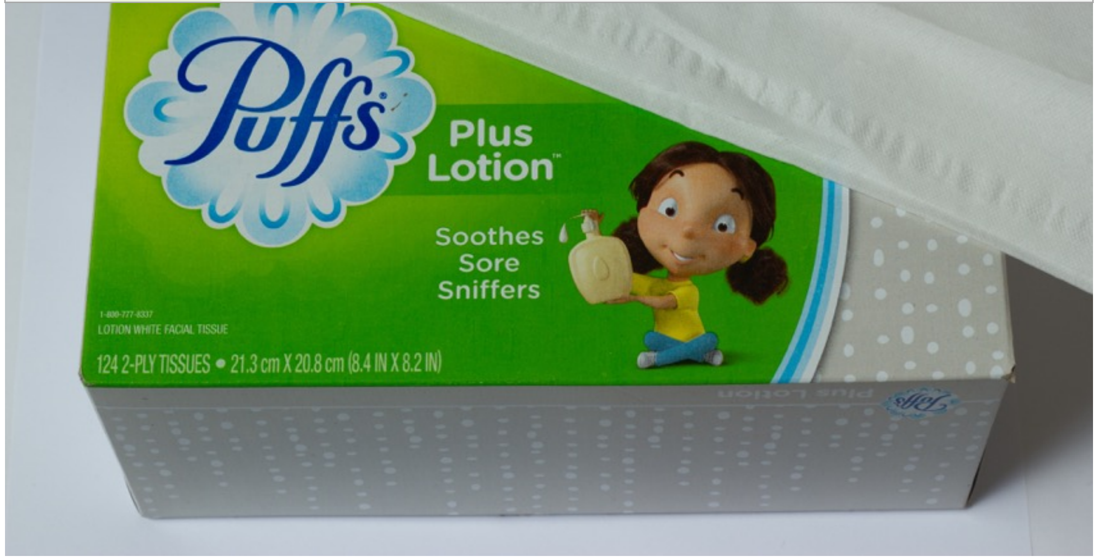

GSM: 42 g/m^2

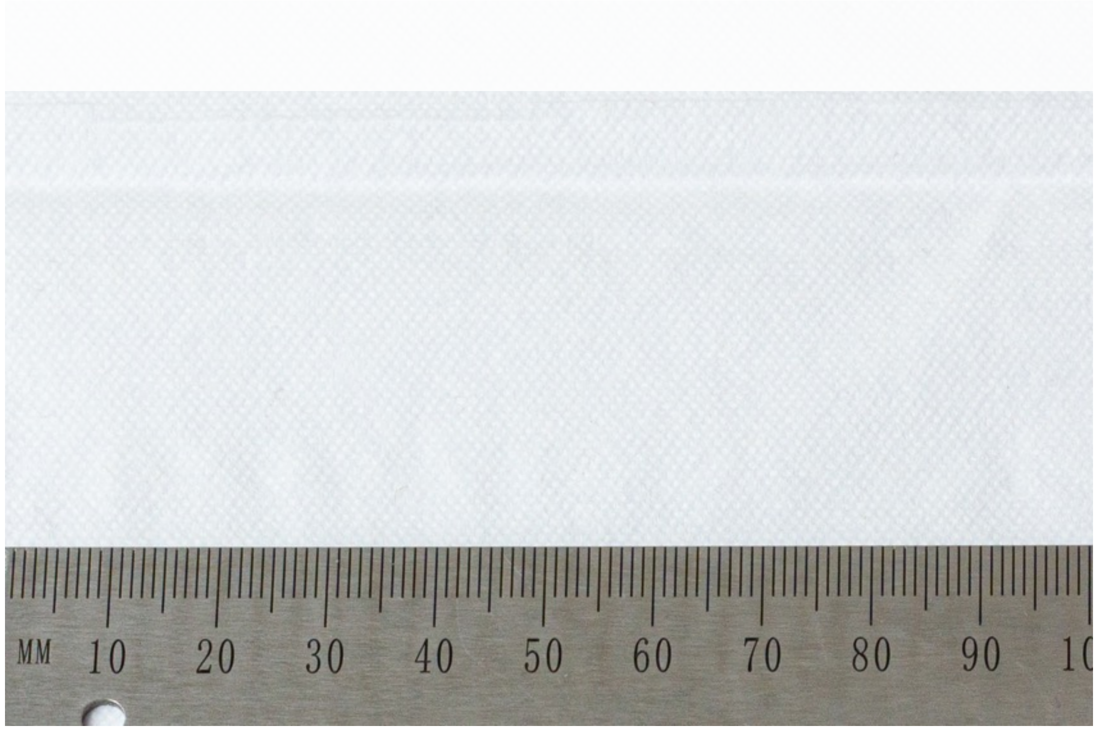

Surgical drape - SMS

Layers	airflow resistance	Filtration efficiency (%)
1	3.83	81.3
2	6.5	94.15
4	10.17	98.61

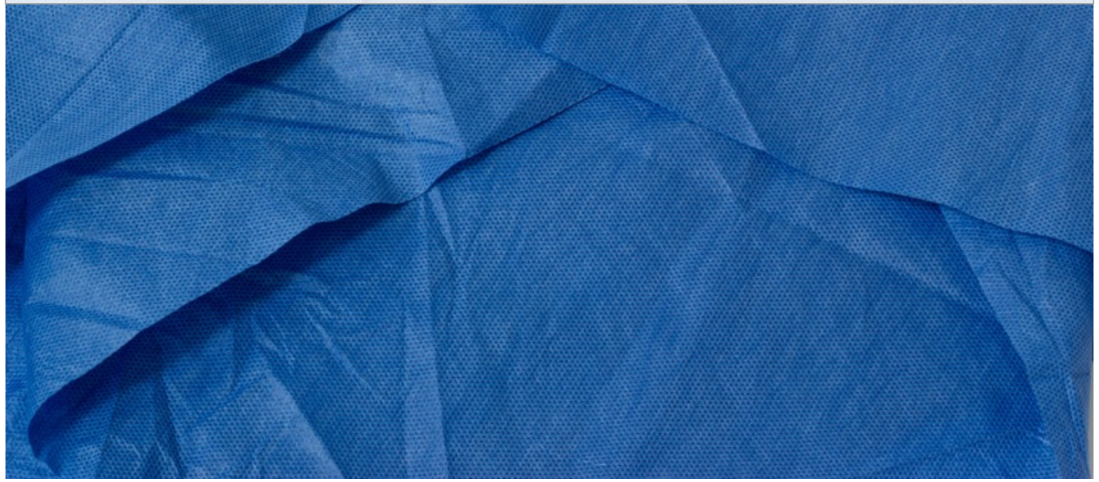

Spunbond Meltblown Spunbond, commonly known as SMS is a tri laminate non-woven fabric.

GSM: 37 g/m^2

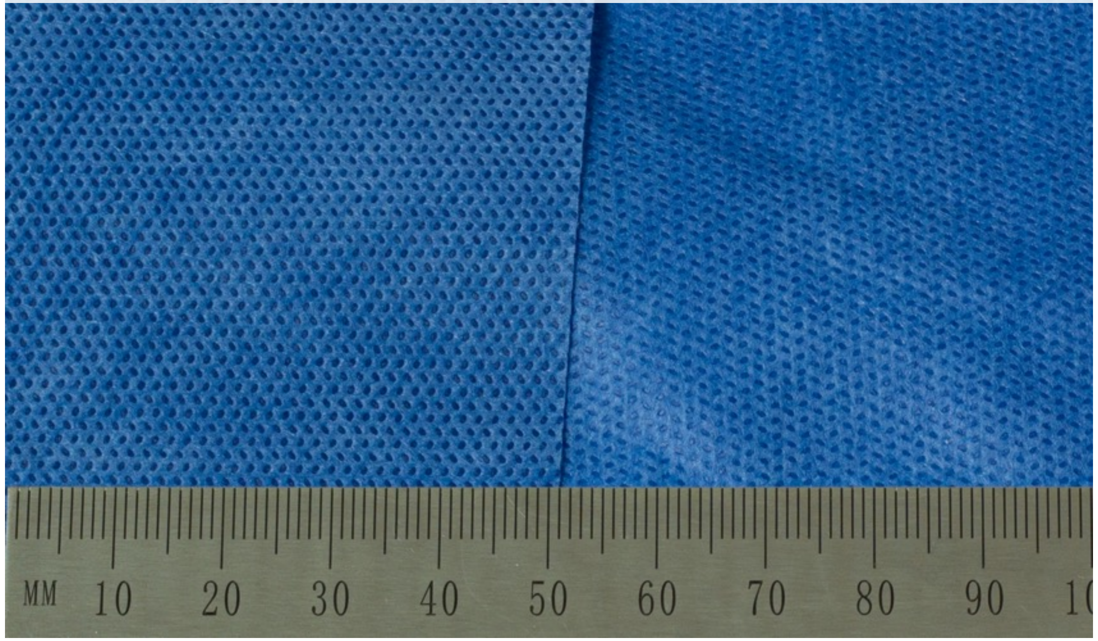

Surgical gown (paper like)

Layers	airflow resistance	Filtration efficiency (%)
1	3.83	79.01

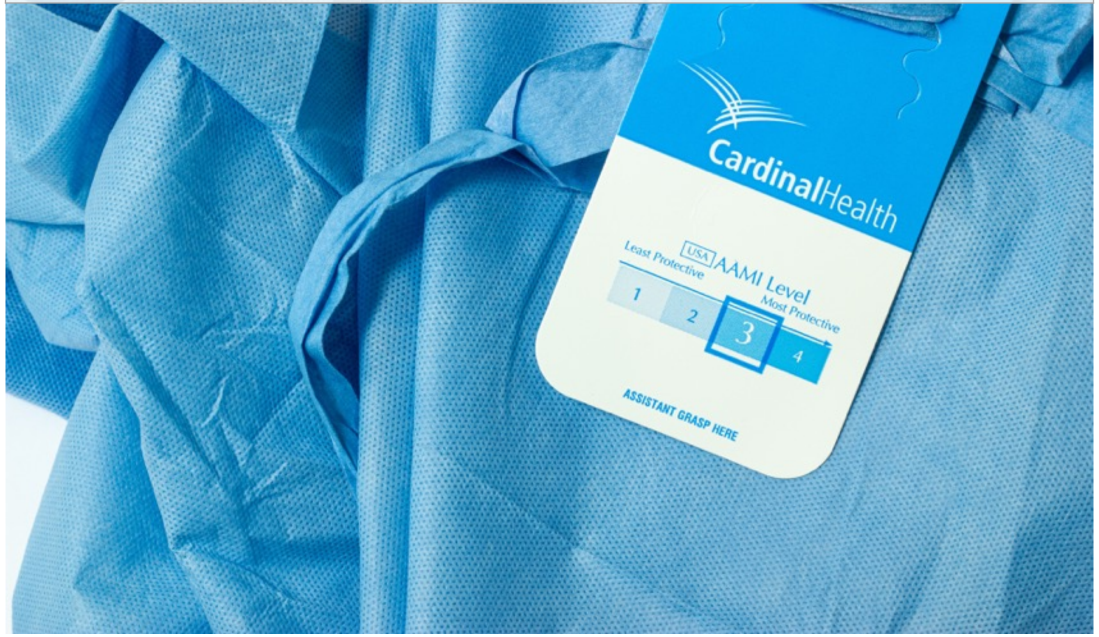

Spunbond Meltblown Spunbond, commonly known as SMS is a tri laminate non-woven fabric. Appears very similar to surgical drape. GSM: 42 g/m^2

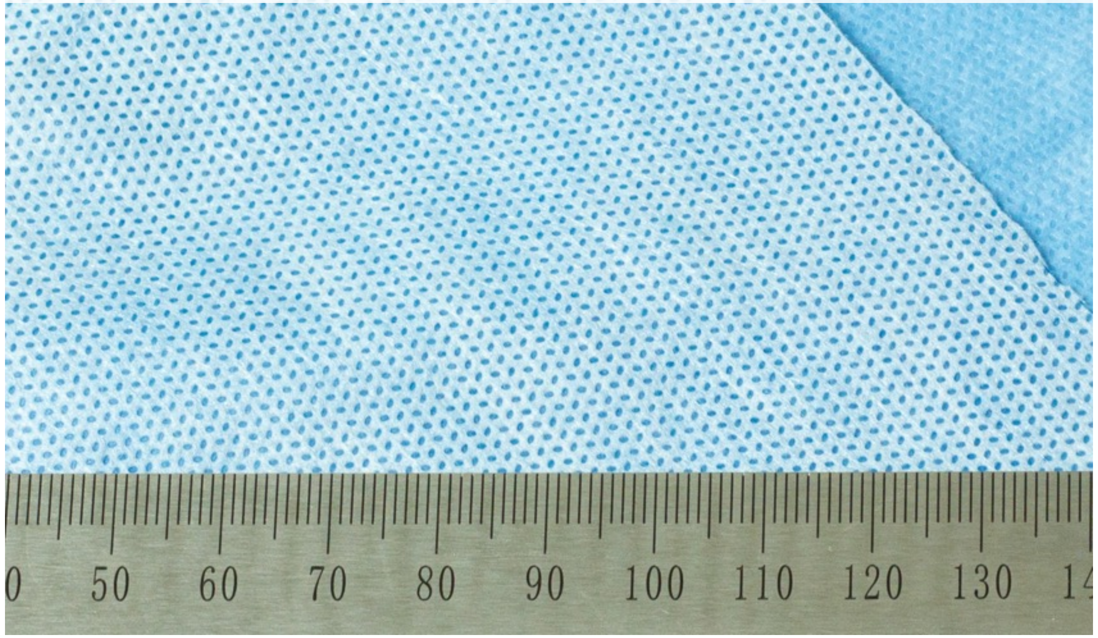

3M N95 respirator

Layers	airflow resistance	Filtration efficiency (%)
Mask wall	5	99.99

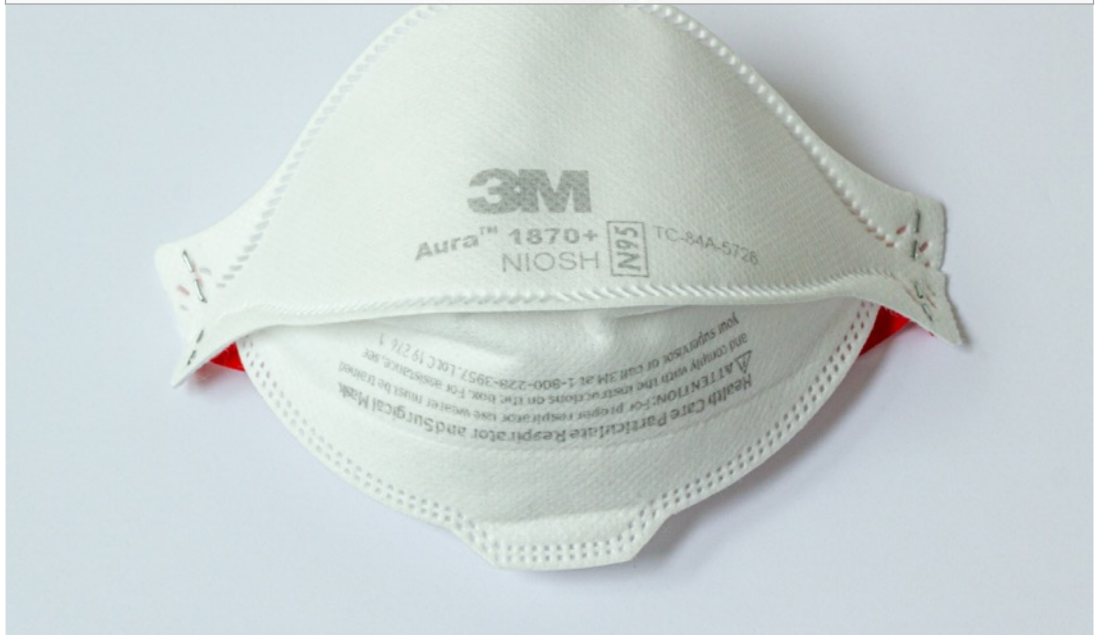

Low airflow resistance.

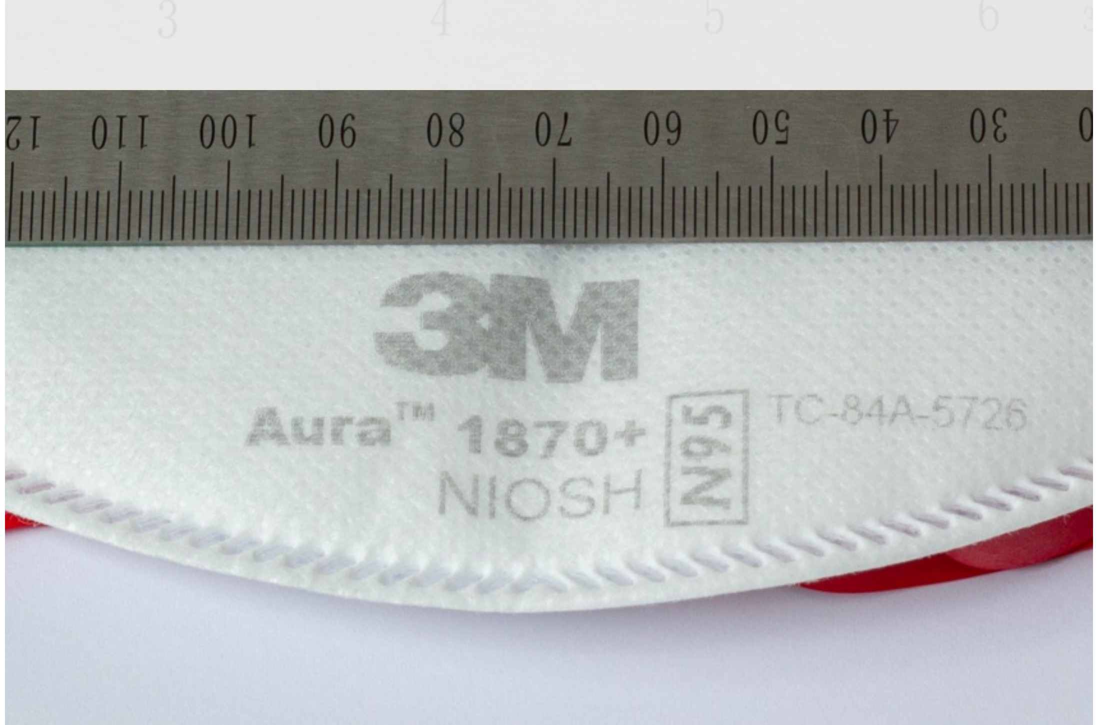

Halyard N95 respirator

**Layers**	**airflow resistance**	**Filtration efficiency (%)**
Mask wall	15.5	99.99

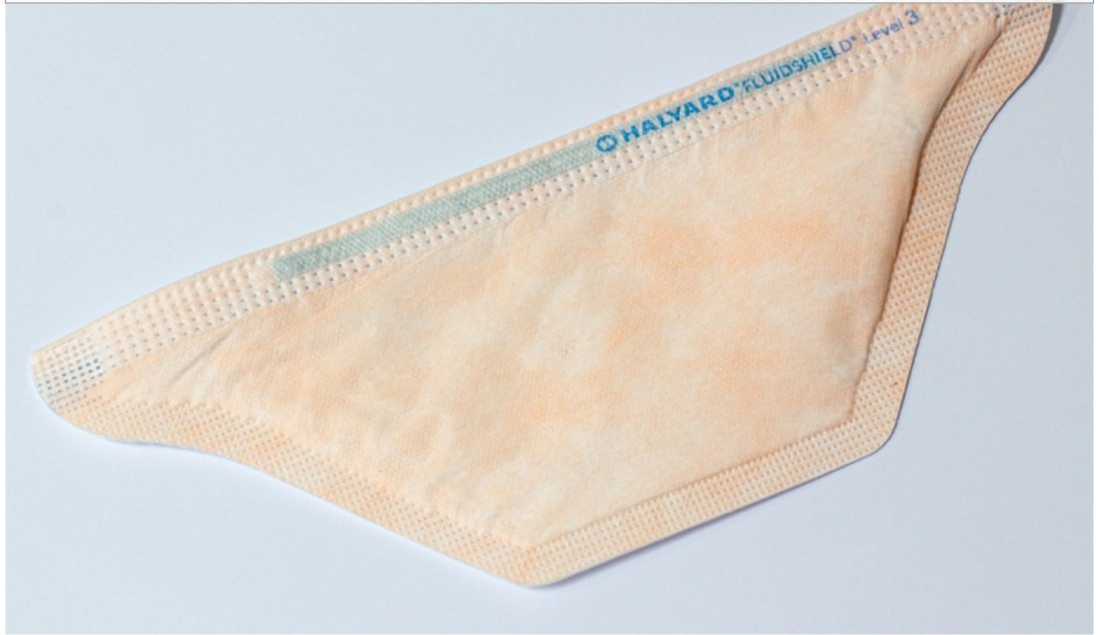

High airflow resistance but large active surface area.

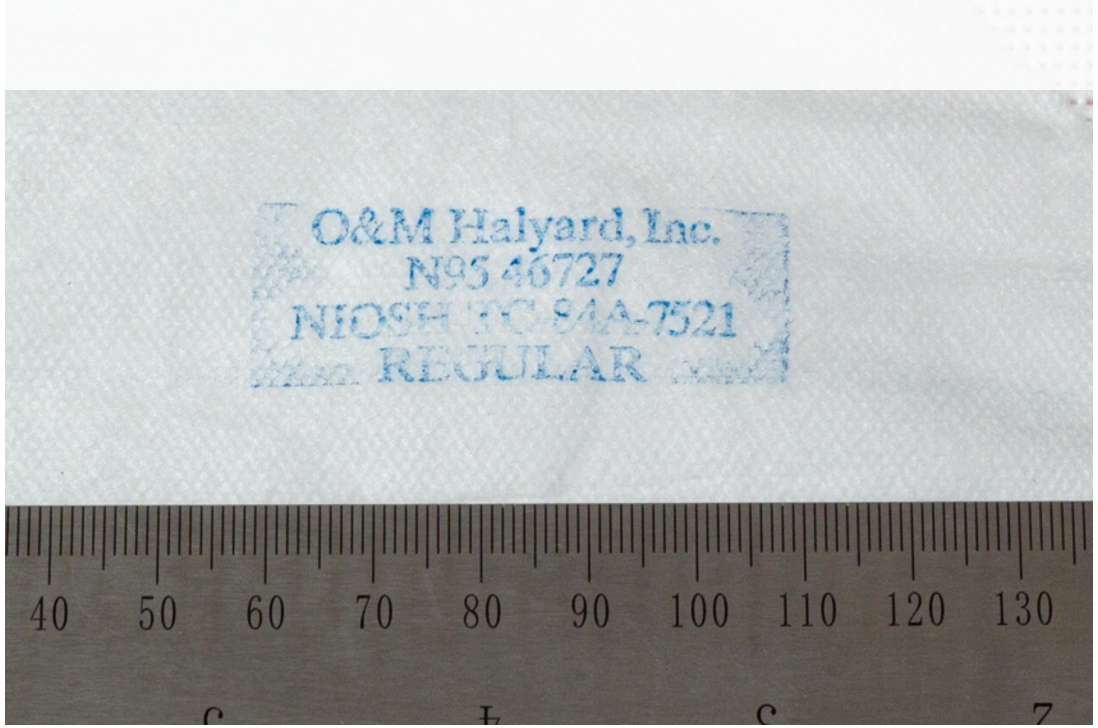

Surgical mask

Layers	airflow resistance	Filtration efficiency (%)
1	7	86.4
2*	12.67	96.81

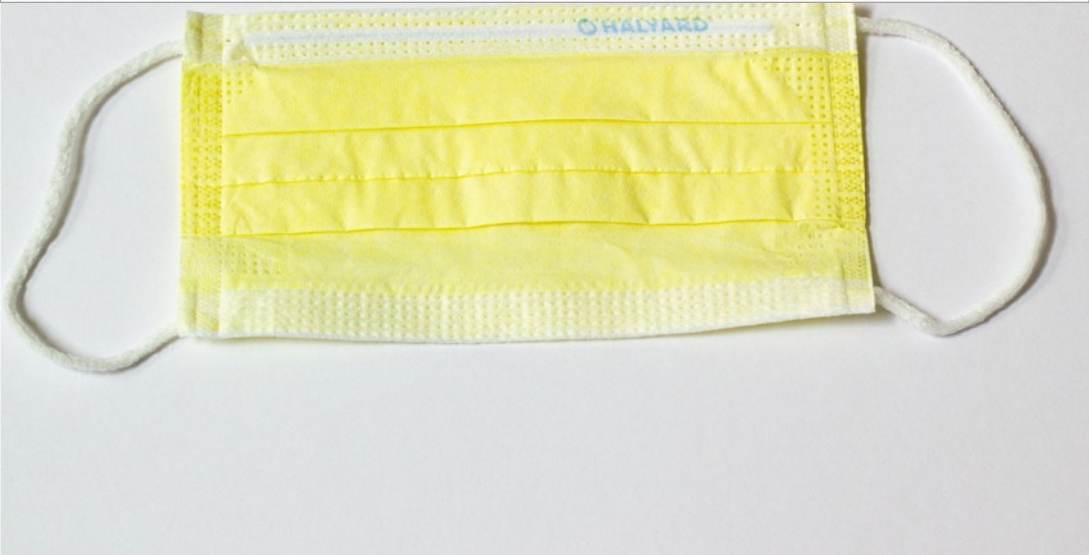

Thin, paper like, non-woven fabric. Layers are folded. Measurement was done on single layer after unfolding the mask. GSM: 58 g/m^2

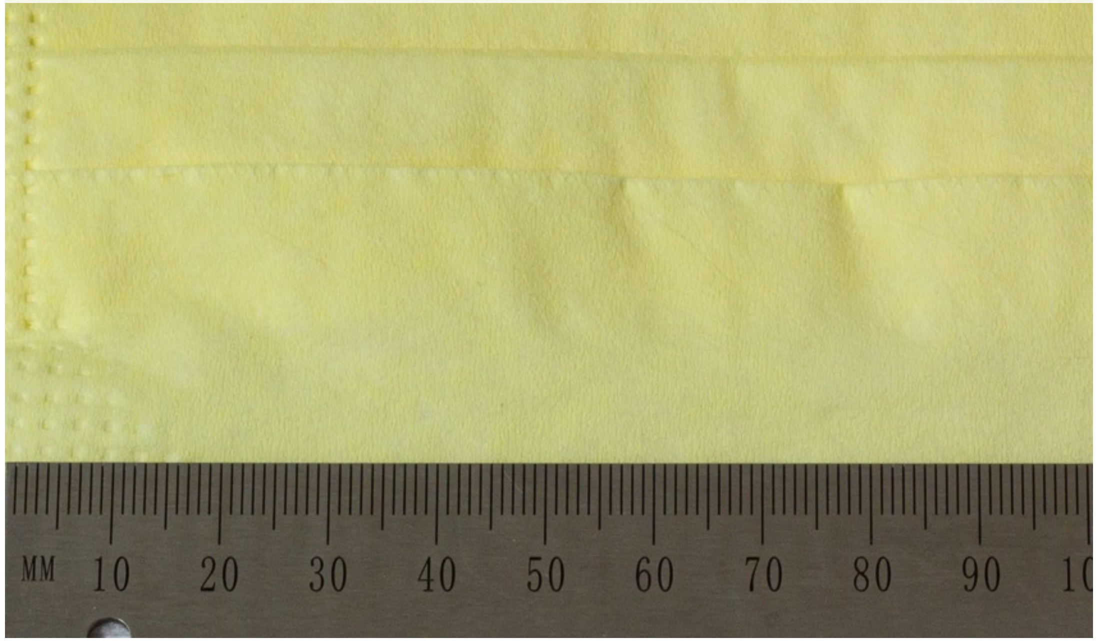

*2 layers (2 masks at a time) are usually not used.

Vacuum cleaner bag – controversial if safe…

Layers	airflow resistance	Filtration efficiency (%)
1*	5.17	98.73

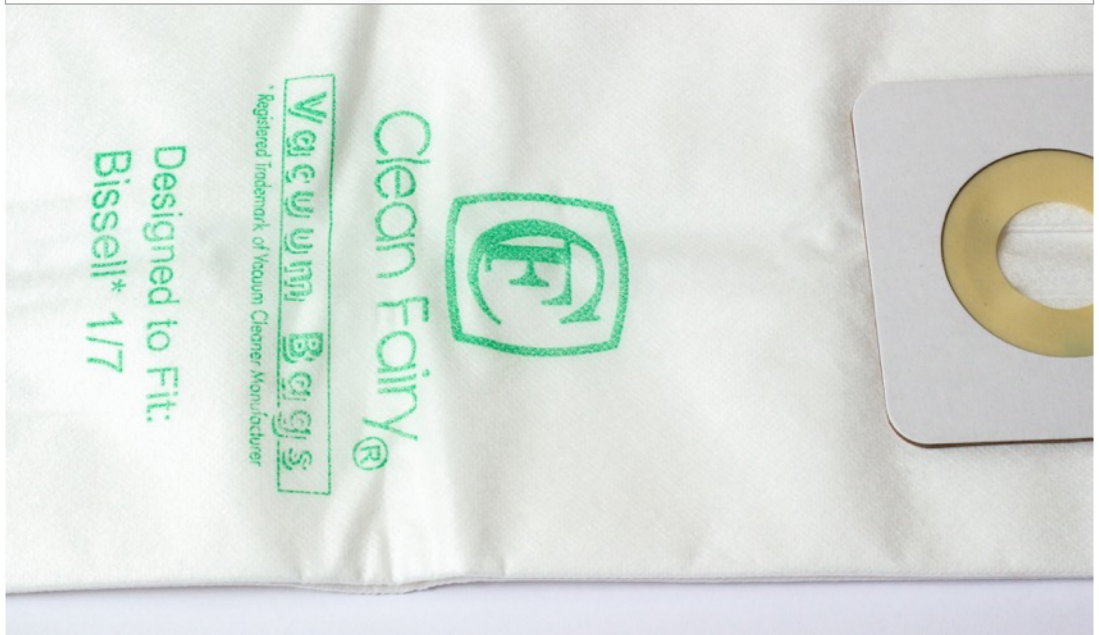

GSM: 125 g/m^2

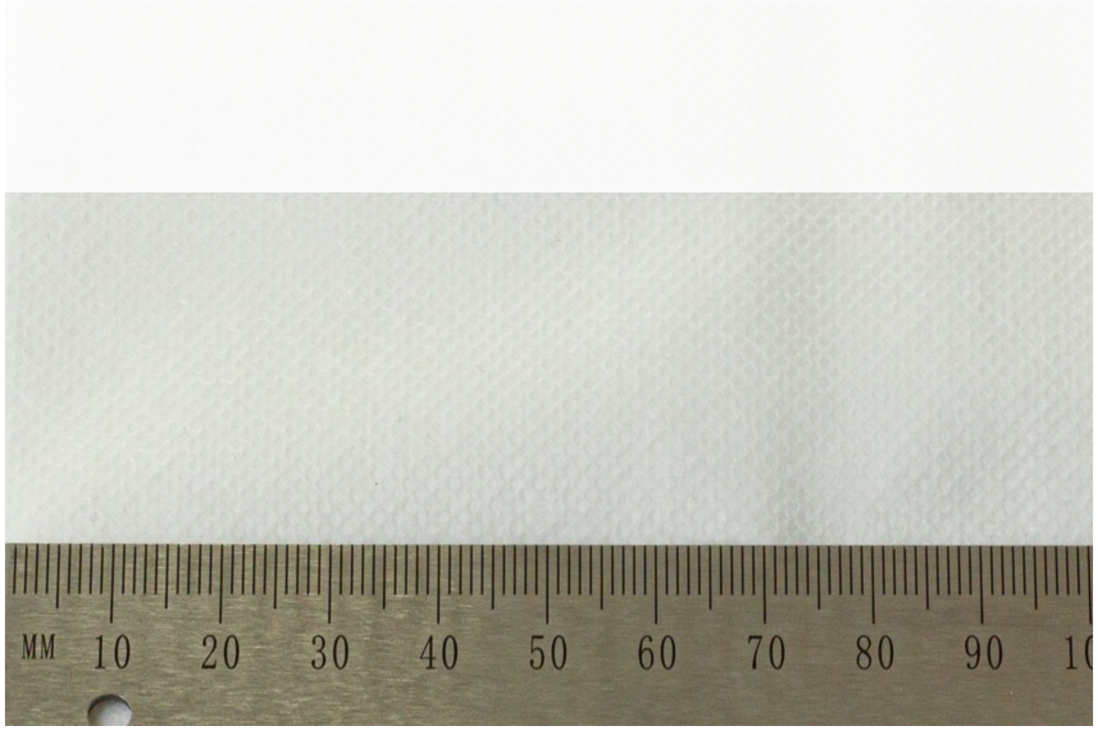

* The wall of the bag contains multiple various layers.

Nothing – negative control

Layers	airflow resistance	Filtration efficiency (%)
0	0	0.84

Both filtration test and airflow resistance test ran with no material inserted.

Filtration performance

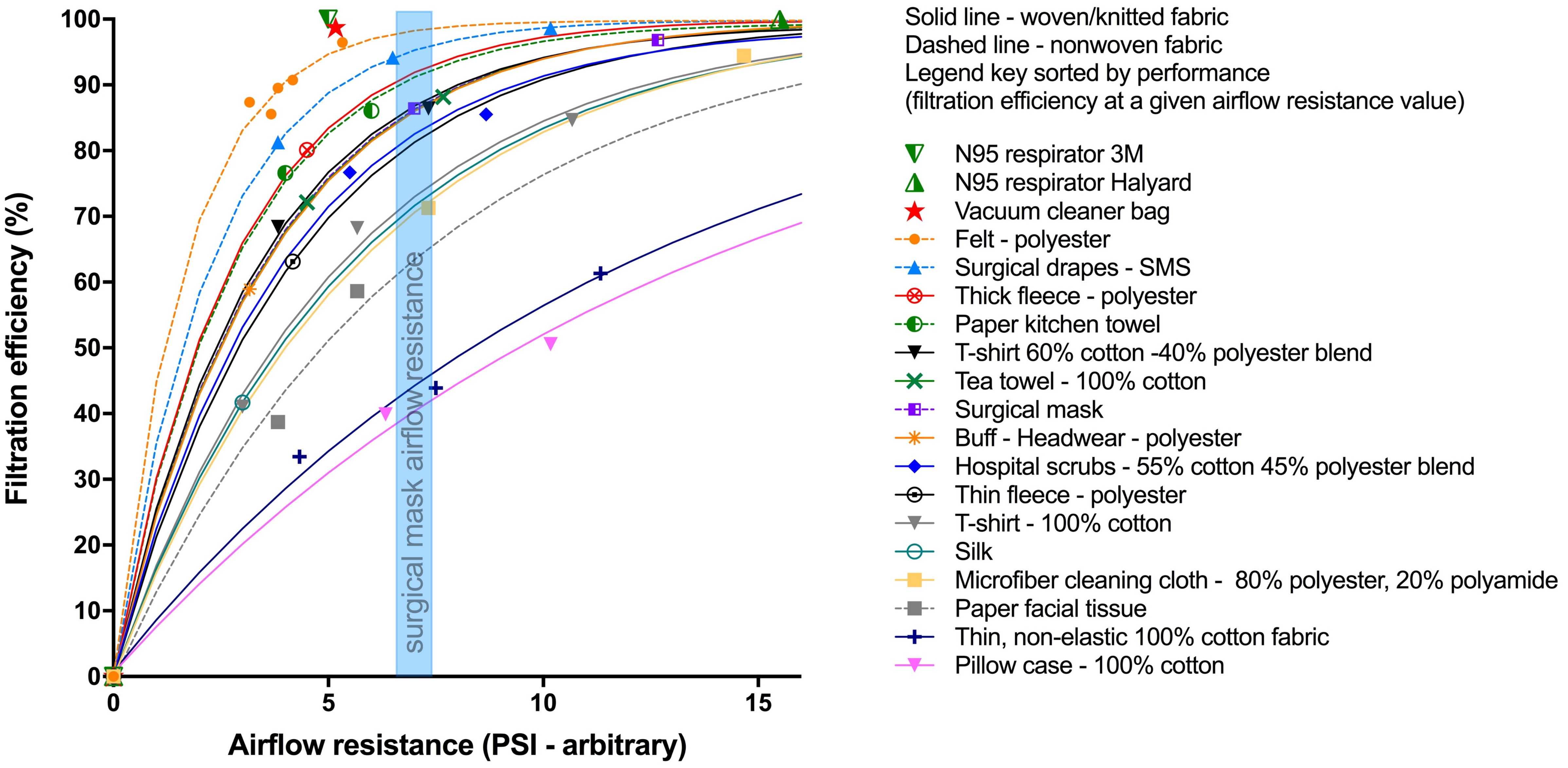

Supplement: Supplementary file 1 [file S1935789320002621sup001.pdf]
